# Endowing Flexibility of Alveolar Type II Cells to Recapitulate Development From Lung Bud to Alveoli

**DOI:** 10.1002/advs.77773

**Published:** 2026-10-04

**Authors:** Hongqian Ma, Yuxin Zhang, Lujia Sun, Hong Jiang, Fan Li, Wenming Liu, Shenao Zhou, Mengting Wang, Weijian Huang, Xu Zhou, Caiyang Chen, Qiuqiu Zhang, Xiaoyan Jin, Feihan Yan, Hongping Wu, Yonghong Yin, Xinling Wang, Hexin Yan, Weifeng Yu

**Affiliations:** ^1^ Department of Anesthesiology, Renji Hospital, Shanghai Jiao Tong University School of Medicine Key Laboratory of Anesthesiology (Shanghai Jiao Tong University) Ministry of Education Shanghai China; ^2^ Shanghai Engineering Research Center of Peri‐operative Organ Support and Function Preservation (20DZ2254200) Shanghai China; ^3^ Shanghai Institute of Infectious Disease and Biosecurity Key Laboratory of Medical Molecular Virology (MOE/NHC/CAMS) School of Basic Medical Sciences Fudan University Shanghai China; ^4^ Department of Thoracic Surgery 905 Hospital of People's Liberation Army Navy Shanghai China; ^5^ Celliver Biotechnology Inc. Shanghai China; ^6^ Duke Kunshan University Kunshan Jiangsu Province China; ^7^ Molecular Epidemiology Laboratory, Eastern Hepatobiliary Surgery Hospital Second Military Medical University Shanghai China

**Keywords:** alveolar organoids, alveolar progenitor‐like cells, influenza A virus, lung regenerative repair

## Abstract

Alveolar type 2 (AT2) cells, which originate from bud tip progenitors during lung development, maintain pulmonary homeostasis through surfactant secretion and regenerative capacity, yet their plasticity has been challenging to model in vitro, limiting their applications in disease modeling and regenerative therapy. Here, a chemically defined reprogramming strategy is developed that enables efficient conversion of adult human AT2 cells into a highly expandable alveolar progenitor‐like state that, at late passages, closely resembles primitive lung bud tip epithelium. These progenitor cells robustly self‐organize into three‐dimensional alveolar progenitor‐like organoids (APLOs), which can be further efficiently differentiated into mature alveolar‐like organoids (ALOs). In vitro, ALOs serve as a physiologically relevant platform for modeling influenza A virus (IAV) infection and mounting innate immune responses characterized by interferon signaling, thereby enabling mechanistic studies of viral pathogenesis and host defense. In vivo, following intratracheal transplantation into lung‐injured mice, APLO‐derived cells exhibit stable engraftment and differentiate into alveolar epithelial lineages, leading to improvement in pulmonary function. In summary, this study establishes an adult‐derived, expandable alveolar progenitor platform generated through chemical reprogramming that recapitulates key aspects of alveolar development, disease modeling, and regenerative potential.

## Graphical abstract

Adult human alveolar type 2 cells are chemically reprogrammed into expandable alveolar progenitor‐like cells that acquire bud tip‐like features during expansion and form organoids that mature into alveolar‐like structures. These organoids model influenza A virus infection and innate immune responses in vitro, while transplanted cells engraft, differentiate into AT2 and AT1 lineages, and improve pulmonary function after lung injury.

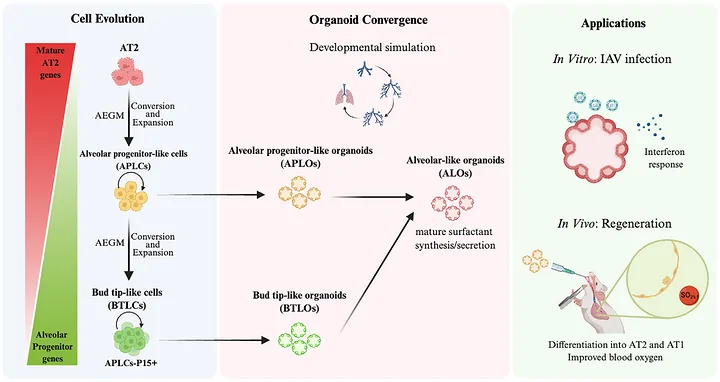

## 1 Introduction

Pulmonary diseases represent the third leading cause of mortality worldwide, underscoring the urgent need to elucidate the mechanisms driving the initiation and progression of respiratory pathologies. Alveolar epithelial injury is a hallmark of diverse lung diseases, including chronic obstructive pulmonary disease (COPD), pulmonary fibrosis [1], and severe infections such as SARS and COVID‐19 [2, 3], contributing significantly to global disease burden. Major advances in understanding human lung diseases, from acute insults to chronic degenerative processes, will depend on the development of physiologically relevant human in vitro systems. It is therefore critical to develop idealized in vitro models that faithfully recapitulate the structure, function, and pathophysiology of the human lung.

AT2 cells serve as stem cells within the alveolar epithelium, capable of self‐renewal and differentiation into AT1 cells to promote repair following lung injury [4, 5]. Organoids derived from primary adult human AT2 cells have been successfully generated and employed to model SARS‐CoV‐2 infection [6, 7, 8, 9, 10, 11]. Furthermore, expanded populations of adult human lung progenitors exhibit the potential to differentiate into AT2 cells [10]. However, the utility of adult AT2 organoids is limited by their slow proliferation rate [6, 7]. An alternative strategy involves deriving AT2‐like cells from pluripotent stem cells (PSC‐iAT2s), which offers advantages in scalability and genetic tractability [12, 13, 14]. Yet PSC differentiation protocols are often lengthy and technically complex [15], and frequently yield heterogeneous cultures with spontaneous off‐target differentiation [14]. Moreover, PSC‐derived systems raise important translational concerns regarding developmental immaturity and safety. Consequently, an alternative source of readily expandable human AT2 cells holds significant value for investigating lung development and disease treatment.

During human lung development, the distal bud epithelium constitutes a multipotent progenitor population that drives branching morphogenesis and distal lineage specification [16, 17]. During the pseudoglandular stage (5‐17 post‐conceptual weeks, pcw), these cells co‐express SOX2 and SOX9. As development proceeds into the canalicular stage (approximately 16–26 pcw), lineage segregation occurs: bud stalk cells become SOX2^+^SOX9^−^ and adopt proximal airway identities, whereas bud tip cells maintain SOX9 expression, downregulate SOX2, and commit toward alveolar fates [18]. From 17 pcw onward, immature AT2 precursors emerge and gradually upregulate canonical AT2 differentiation programs [19, 20]. Studies have shown that fetal bud tip–derived organoids can be expanded and differentiated into AT2 cells, providing powerful developmental models [19, 21]. Nevertheless, restricted accessibility of fetal tissue and ethical constraints fundamentally limit their broader application.

Here, we established a chemically defined reprogramming strategy that unlocks the intrinsic plasticity of adult human AT2 cells, enabling their long‐term expansion through a dedifferentiated progenitor‐like state. We demonstrate that mature AT2 cells can be induced into highly proliferative alveolar progenitor‐like cells (APLCs), which progressively downregulate mature AT2 programs while acquiring progenitor‐associated transcriptional features. Remarkably, after extended passaging, these cells stabilize into a SOX9^+^ bud tip–like state closely resembling fetal distal epithelial progenitors, which we define as bud tip‐like cells (BTLCs). Both APLCs and BTLCs exhibit robust organoid‐forming capacity and can be efficiently redifferentiated into mature alveolar‐like organoids (ALOs) exhibiting mature AT2 features together with an AT1‐associated cell state.

Collectively, this work establishes an adult‐derived, expandable alveolar progenitor platform that captures bidirectional epithelial state transitions along the developmental axis. This system provides a powerful foundation for modeling human alveolar development and lung disease, and enabling regenerative therapeutic strategies for distal lung disorders.

## 2 Results

### 2.1 Establishing a Chemically Defined Condition for Long‐Term and Large‐Scale Expansion of Alveolar Epithelial Cells in Vitro

Currently, the long‐term expansion and maintenance of functional AT2 cells in vitro pose a significant challenge, limiting their utility for disease modeling and regenerative applications. To address this issue, we adapted a chemical reprogramming strategy modified from which was previously developed for hepatocytes [22] to induce a progenitor‐like state in primary adult human AT2 cells. Briefly, single‐cell dissociated hAT2 cells derived from distal parenchymal regions of healthy donor lungs were isolated by fluorescence‐activated cell sorting (FACS) for the hAT2 cell surface marker HTII‐280 (CD31^−^CD45^−^EPCAM^+^HTII‐280^+^) (Figure 1A and Figure S1A) [5, 23].

**FIGURE 1:**
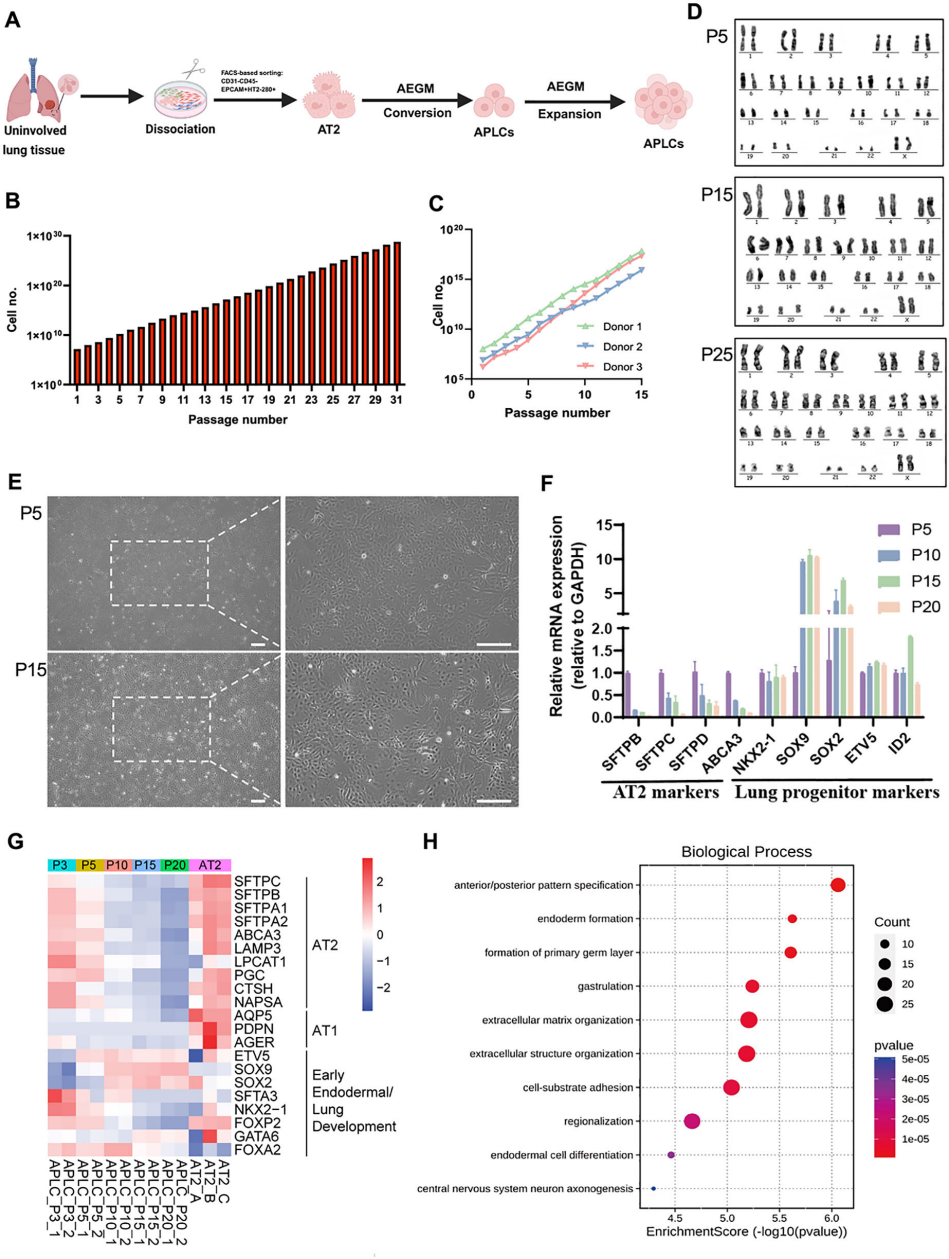
Generation of alveolar epithelial cell‐derived alveolar progenitor‐like cells in vitro. (A) Schematic diagram of the protocol for converting primary human AT2 cells into APLCs. Created in BioRender. Ma, H. (2026) https://BioRender.com/nebp12r (B) Growth curve of APLCs cultured in AEGM. (C) Growth curve of APLCs derived from three independent donors. (D) Representative chromosome karyotype analysis of APLCs at passages 5, 15, and 25 (P5, P15, and P25). (E) Bright‐field microscopy images depicting the morphology of APLCs at early passage (P5) and late passage (P15). Scale bars, 200 µm. (F) Quantitative PCR (qPCR) analysis of AT2 cell markers and lung progenitor cell markers in APLCs across different passages. Data represent mean ± SD (n = 3 independent experiments). (G) Heatmap depicting the relative expression levels (log2 fold change) of key alveolar epithelial‐ and lung lineage‐associated genes in APLCs and primary AT2 at different passage numbers. (H) Significantly upregulated signaling pathways in late‐passage (P15) APLCs compared to primary hAT2 cells, identified by GO enrichment analysis.

When cultured in an optimized Alveolar Epithelial Growth Medium (AEGM), supplemented with CHIR99021, Y27632, A83‐01, FGF10, and EGF, hAT2 cells exhibited sustained proliferative capacity and were passaged continuously for more than 30 generations (Figure 1B). This vigorous expansion was consistently reproduced across multiple independent donors (Figure 1C). During serial planar culture, the expanded alveolar epithelial cells were routinely cryopreserved and subsequently thawed. Following recovery, the cells readily reattached and resumed expansion under AEGM conditions, demonstrating their compatibility with routine cryopreservation and long‐term storage. Throughout this long‐term expansion period, cells maintained a stable epithelial morphology, and karyotype analysis revealed no detectable chromosomal abnormalities even up to passage 25, indicating genomic stability during long‐term culture (Figure 1D,E, and Figure S1B).

To evaluate the cellular identity during this serial passaging, we performed quantitative PCR (qPCR) and bulk RNA sequencing analyses. While early‐passage cells highly expressed canonical AT2 markers (including SFTPB, SFTPC, SFTPD, and ABCA3), extended culture revealed a progressive decline in these AT2‐associated genes. This was accompanied by a concomitant upregulation of progenitor‐associated transcription factors, including SOX9, SOX2, ETV5, and ID2, which reached a stable plateau by passage 15 (Figure 1F,G, and Figure S1C). In contrast, markers of basal (KRT5, TP63), secretory (SCGB1A1/CC10), and ciliated (FOXJ1) epithelial lineages remained undetectable throughout the culture period (Figure S1,C,D). The absence of these lineage‐specific markers indicates that the long‐term expanded cells are neither derived from basal cells or other proximal airway epithelial lineages, nor do they differentiate toward these lineages or exhibit detectable contamination during in vitro culture. Finally, pathway enrichment analysis further demonstrated that genes associated with early alveolar epithelial progenitor development were significantly upregulated in these cells compared with primary hAT2 cells (Figure 1H).

Collectively, these findings indicate that hAT2 cells undergo dedifferentiation toward a progenitor‐like state under AEGM culture conditions. We therefore designate these chemically reprogrammed and expandable cells as alveolar progenitor‐like cells (APLCs), which provide a stable and scalable planar epithelial cell source for further applications.

### 2.2 Single‐Cell RNA Sequencing Further Defines the Characteristics of Alveolar Epithelial‐Derived Progenitor‐Like Cells

To further elucidate the changes in cell subpopulations during passaging, we performed single‐cell RNA sequencing (scRNA‐seq) on early‐passage (P5) and late‐passage (P15) APLCs cultured in AEGM (Figure 2A). The resulting merged uniform manifold approximation and projection (UMAP) plots showed these epithelial cells were divided into multiple clusters, including AT2 (cluster 0,1,4), AT1(cluster 6), bud tip progenitor cells (BTPCs) (cluster 2,9), proliferating AT2 (cluster 3,8), proliferating BTPCs (cluster 5) and a population of transient cells (cluster 7) (Figure 2,B,C). The AT2 clusters were characterized by high expression of canonical AT2 markers, including surfactant proteins (SFTPA, SFTPB, SFTPC, and SFTPD) and associated genes (ABCA3, LAMP3, NAPSA, and LPCAT1). These AT2 cells predominated in early‐passage APLCs but exhibited a marked reduction in relative abundance with extended passaging (Figure 2,D,E, and Figure S2A). In contrast, the BTPC clusters expressed key progenitor‐associated transcription factors, including SOX9, SOX2, and ETV5. The proportion of BTPCs progressively increased over time, ultimately becoming the dominant population in late‐passage APLCs (Figure 2,D,E, and Figure S2B). Based on their stable progenitor transcriptional profile and close resemblance to developmental lung bud tip cells, we designated this late‐passage, bud tip–like APLC population as bud tip–like cells (BTLCs).

**FIGURE 2:**
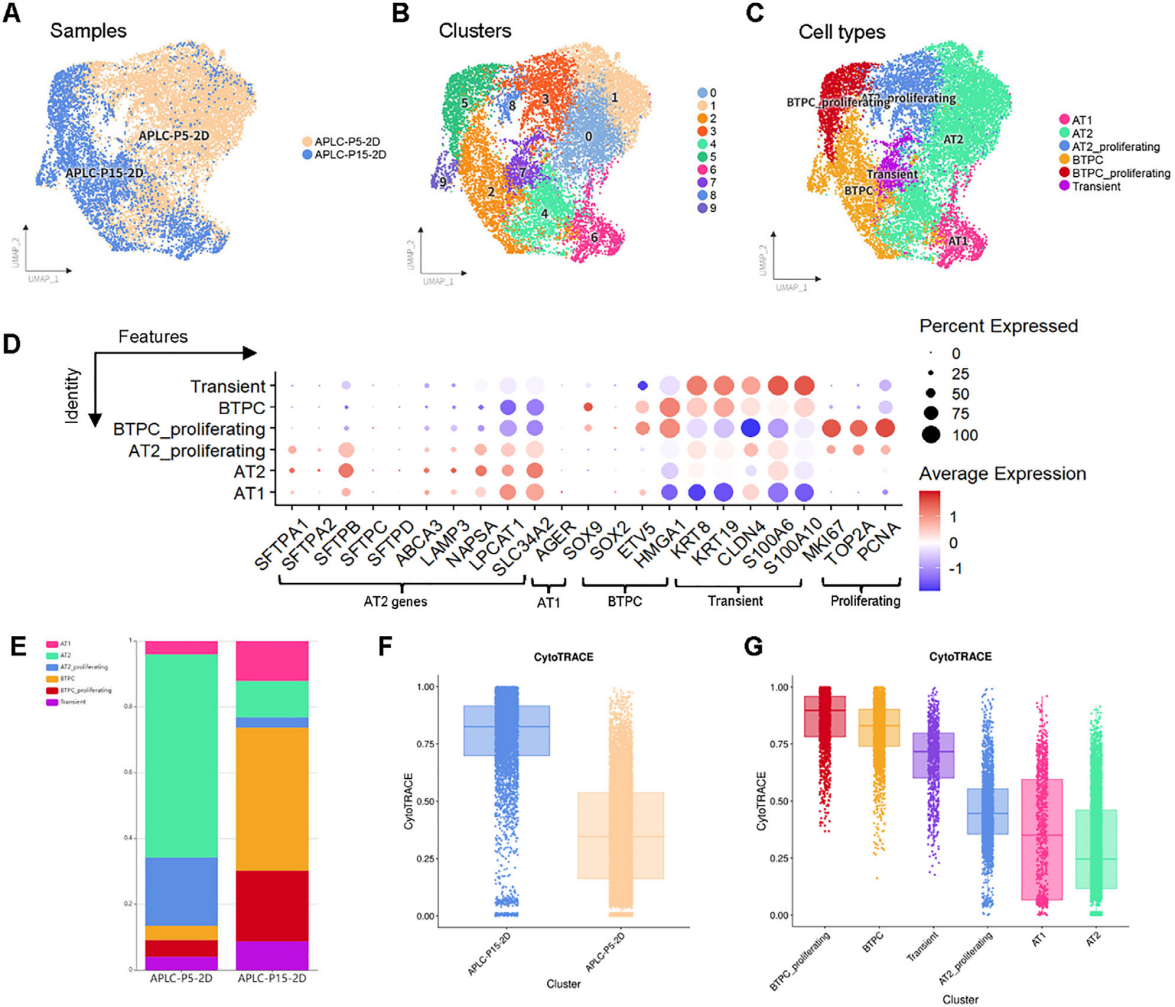
Single‐cell RNA sequencing characterization of alveolar epithelial progenitor cells (APLCs). (A) Uniform Manifold Approximation and Projection (UMAP) visualization of 14216 single‐cell transcriptomes from APLCs at passage 5 (P5) and passage 15 (P15). (B) UMAP plot depicting distinct cell clusters identified within the APLC cultures. (C) UMAP visualization colored by cell types. (D) Dot plot describing the expression levels of key genes associated with AT2 identity, AT1 identity, lung bud tip progenitor cell, transient state, and cellular proliferation across the identified APLC clusters. Dot size represents the percentage of cells expressing the gene within a cluster; color intensity indicates the average expression level. (E) Compositional analysis showing the relative proportions of identified cell clusters in APLCs at P5 versus P15. (F) Evaluation of stemness properties across the entire APLC population at P5 and P15 using a curated stemness gene signature. (G) Comparative stemness assessment of individual cell clusters identified within APLC cultures.

Subsets of both AT2 and BTPC populations co‐expressed proliferation‐associated markers (MKI67, TOP2A, and PCNA), indicating the presence of actively cycling subpopulations within each lineage. A small number of AT1 cells expressing AGER were also detected, consistent with previous observations of spontaneous AT2‐to‐AT1 differentiation under two‐dimensional culture conditions. In addition, a minor cluster of transitional epithelial cells was identified, characterized by the expression of KRT8, KRT19, CLDN4, S100A6, and S100A10, markers previously associated with intermediate states during AT2‐to‐AT1 differentiation (Figure 2D).

Importantly, throughout the entire passaging process, no expression of basal cell (TP63), ciliated cell (FOXJ1), or secretory cell (SCGB3A2) markers was detected, indicating the absence of alternative airway epithelial lineage differentiation (Figure S2C). Furthermore, CytoTRACE analysis revealed a progressive increase in cellular stemness potential from early to late passages, consistent with a shift toward a progenitor‐dominated cellular state (Figure 2,F,G).

Collectively, these data demonstrate a gradual and unidirectional transition of AT2 cells toward a progenitor‐oriented transcriptional state under AEGM culture conditions, further validating the progenitor‐like identity and cellular plasticity of APLCs.

### 2.3 Generation of Alveolar Progenitor‐Like Organoids, Bud Tip‐Like Organoids and Alveolar‐Like Organoids

Following the establishment of a stable planar progenitor cell source, we next assessed the capacity of APLCs to form three‐dimensional (3D) organoids. Cells were transferred to suspension culture in non‐attachment plates supplemented with diluted Matrigel, a culture condition previously reported to support alveolar differentiation [24] (Figure 3A). Under these conditions, APLCs spontaneously self‐organized into 3D epithelial structures displaying marked heterogeneity in size and morphology, ranging from complex folded architectures to simple cystic organoids (Figure S3A). Immunofluorescence staining and western blot analysis revealed that organoids derived from early‐passage APLCs (P5) contained only a small fraction of SOX9^+^ cells with low overall SOX9 expression, whereas organoids generated from BTLCs (P15) exhibited a prominent and expanded SOX9^+^ population. (Figure 3,B,F). Notably, expression of canonical AT2 markers was already reduced in APLC‐derived organoids, consistent with the dedifferentiated phenotype observed in planar APLC cultures. This reciprocal pattern of SOX9 acquisition and AT2 marker loss supports a progressive dedifferentiation trajectory during extended culture (Figure 3F). Based on these molecular features, we classified P5 APLC‐derived organoids as alveolar progenitor‐like organoids (APLOs), whereas P15 BTLC‐derived organoids, exhibiting a stable SOX9^+^ bud tip progenitor‐like profile, were designated as bud tip‐like organoids (BTLOs).

**FIGURE 3:**
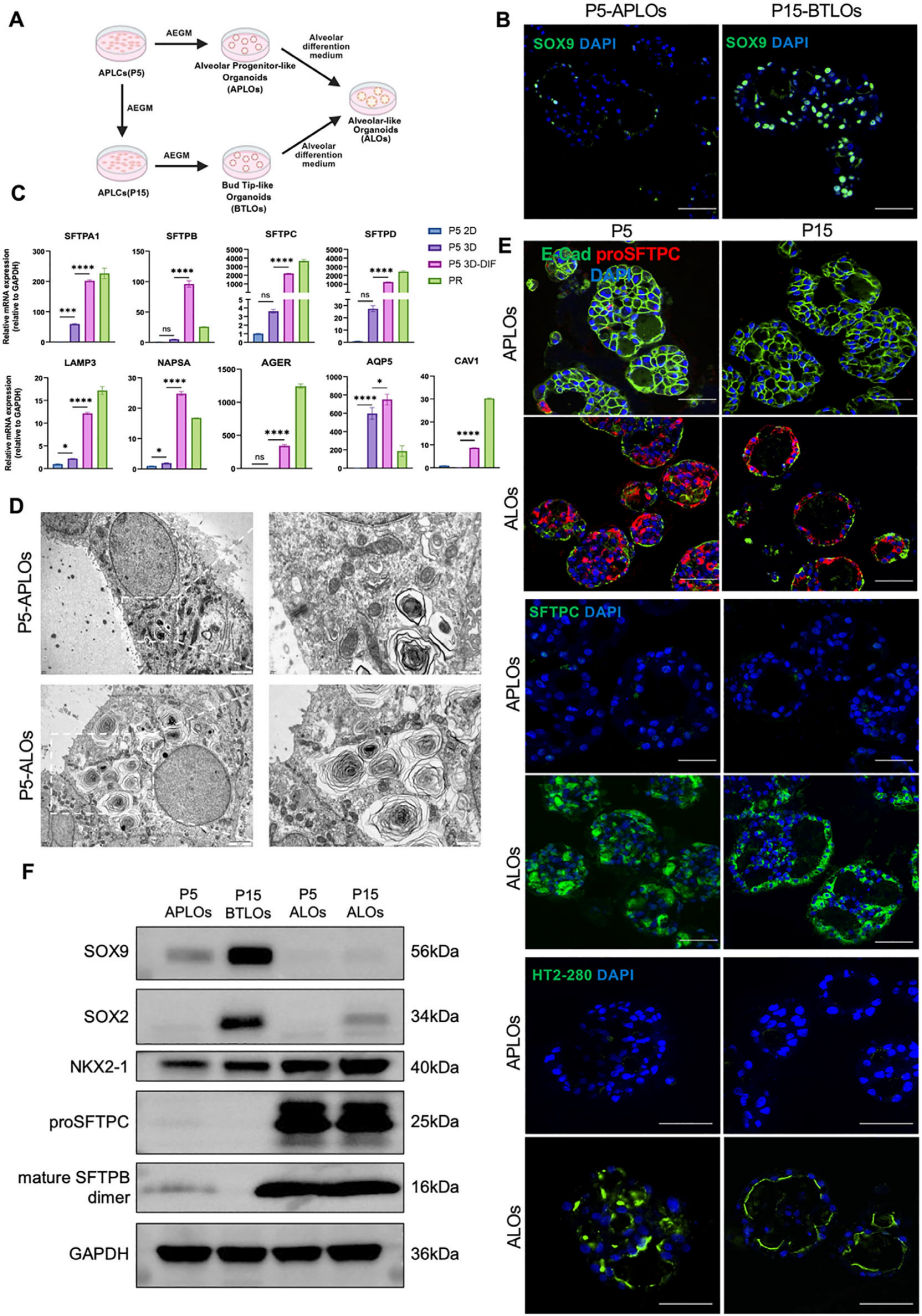
Establishment and characterization of alveolar progenitor‐like organoids (APLOs), bud tip‐like organoids (BTLOs), and alveolar‐like organoids (ALOs). (A) Schematic diagram illustrating the differentiation protocol from APLCs to APLOs, BTLOs, and ALOs. Created in BioRender. Ma, H. (2026) https://BioRender.com/91lb473 (B) Representative immunofluorescence (IF) images showing expression of the bud tip marker SOX9 in P5‐APLOs and P15‐BTLOs. Scale bars, 50 µm. (C) Quantitative PCR (qPCR) analysis of AT2 and AT1 cell marker expression in APLC‐P5‐2D, P5‐APLOs, P5‐ALOs, and human lung tissue. Data are presented as mean ± SD from three independent experimental replicates using samples derived from a single donor (n = 3). Significance was evaluated by one‐way ANOVA with Tukey's multiple comparisons test; ns: not significant, *p < 0.05, **p < 0.01, ***p < 0.001, ****p < 0.0001. See also Supplementary Figure S3B,C. **(D)** Representative transmission electron microscopy (TEM) images of APLOs and ALOs with lamellar body structures. Scale bars, 2 µm (left), 500 nm (upper right), 1 µm (lower right). **(E)** Immunofluorescence staining of the pan‐epithelial marker E‐cadherin and AT2 markers (proSFTPC, SFTPC, and HT2‐280) in APLOs, BTLOs, and ALOs. Scale bars, 50 µm. **(F)** Western blot analysis of bud tip markers (SOX9, SOX2), AT2 markers (proSFTPC, SFTPB dimer), and the lung lineage marker NKX2‐1 in APLOs, BTLOs, and ALOs.

To evaluate their differentiation potential, APLOs and BTLOs were subsequently cultured in alveolar differentiation medium previously shown to induce AT2‐containing alveolospheres from hiPSC‐derived lung progenitors [12, 15]. Under these conditions, both organoid types underwent robust maturation, accompanied by marked upregulation of mature AT2 markers (SFTPA, SFTPB, SFTPC, SFTPD, LAMP3, NAPSA, and HTII‐280) as well as AT1 markers (AGER, AQP5, and CAV1), reaching expression levels comparable to those observed in primary human lung tissue (Figure 3C and Figure S3,B–D).

Transmission electron microscopy (TEM) analysis demonstrated a substantial increase in lamellar body formation following differentiation, indicating acquisition of ultrastructural features characteristic of functional AT2 cells (Figure 3D and Figure S3F). Consistently, immunofluorescence staining showed that undifferentiated APLOs and BTLOs lacked expression of AT2 markers (proSFTPC, SFTPC, and HTII‐280), whereas differentiated organoids displayed strong expression of these markers along with proper basolateral localization of E‐cadherin, consistent with progenitor‐to‐AT2 maturation (Figure 3E). To further evaluate the functional capacity of AT2 cells in differentiated organoids, we examined the uptake and intracellular localization of fluorescently labeled phosphatidylcholine. LysoTracker Red staining revealed numerous discrete acidic vesicular structures within the cytoplasm. Following incubation with β‐BODIPY FL C12‐HPC, the fluorescent phospholipid was readily internalized and appeared as intracellular puncta [24, 25]. A proportion of the BODIPY‐positive signals overlapped with LysoTracker‐positive compartments, whereas additional signals remained spatially distinct (Figure S3E). These findings indicate that AT2‐like cells within the organoids are capable of phospholipid uptake and subsequent trafficking to acidic, lamellar body‐like compartments, supporting their functional capacity for surfactant‐associated lipid handling. Differentiated organoids also exhibited robust uptake of LysoTracker, a fluorescent dye labeling acidic organelles such as lamellar bodies, further supporting functional surfactant‐producing capacity (Figure S3E). Western blot analysis confirmed the dynamic regulation of lineage‐specific markers before and after differentiation (Figure 3F).

We therefore termed these mature structures alveolar‐like organoids (ALOs), which exhibit key phenotypic and functional features of mature AT2 cells, including lamellar body formation and surfactant production and secretion, while also giving rise to cells with some AT1‐associated features. Notably, both APLOs and BTLOs efficiently differentiated into ALOs, with progenitor marker expression diminishing upon maturation, while expression of the lung lineage transcription factor NKX2‐1 was maintained throughout differentiation (Figure S3G). These findings indicate that ALOs represent a differentiated alveolar epithelial state derived from chemically expanded progenitor populations such as APLOs and BTLOs.

### 2.4 Single Cell RNA Sequencing Reveals Alveolar Cell Dedifferentiation and Redifferentiation Processes

To further delineate the cellular composition and dynamic transitions within APLCs during organoid formation and differentiation, we performed scRNA‐seq on APLOs (P5‐3D), BTLOs (P15‐3D), and ALOs (P5‐3D‐Dif and P15‐3D‐Dif) (Figure 4A). Unsupervised clustering analysis revealed that APLOs, BTLOs, and ALOs were composed of four major epithelial populations: AT2 cells, AT1 cells, bud tip progenitor cells (BTPCs), and proliferating BTPCs (Figure 4B). Comparative analysis demonstrated that BTLOs were predominantly enriched for BTPCs, characterized by high expression of canonical bud tip progenitor markers, including SOX9, SOX2, and ETV5. In contrast, APLOs retained a higher proportion of AT2 cells, defined by expression of surfactant protein genes together with SLC34A2, LAMP3, NAPSA, and ABCA3. Consistent with their progenitor‐dominant identity, BTLOs exhibited a marked reduction in AT2 cell representation (Figure 4,C,D). Upon differentiation into ALOs, both APLO‐ and BTLO‐derived organoids displayed a substantial increase in AT2 and AT1 populations, with AT1 cells expressing AGER, CAV1, and AQP5. Proliferating BTPCs, identified by co‐expression of MKI67, TOP2A, and PCNA, were largely restricted to undifferentiated organoids and were markedly diminished following differentiation (Figure 4,C,D, and I).

**FIGURE 4:**
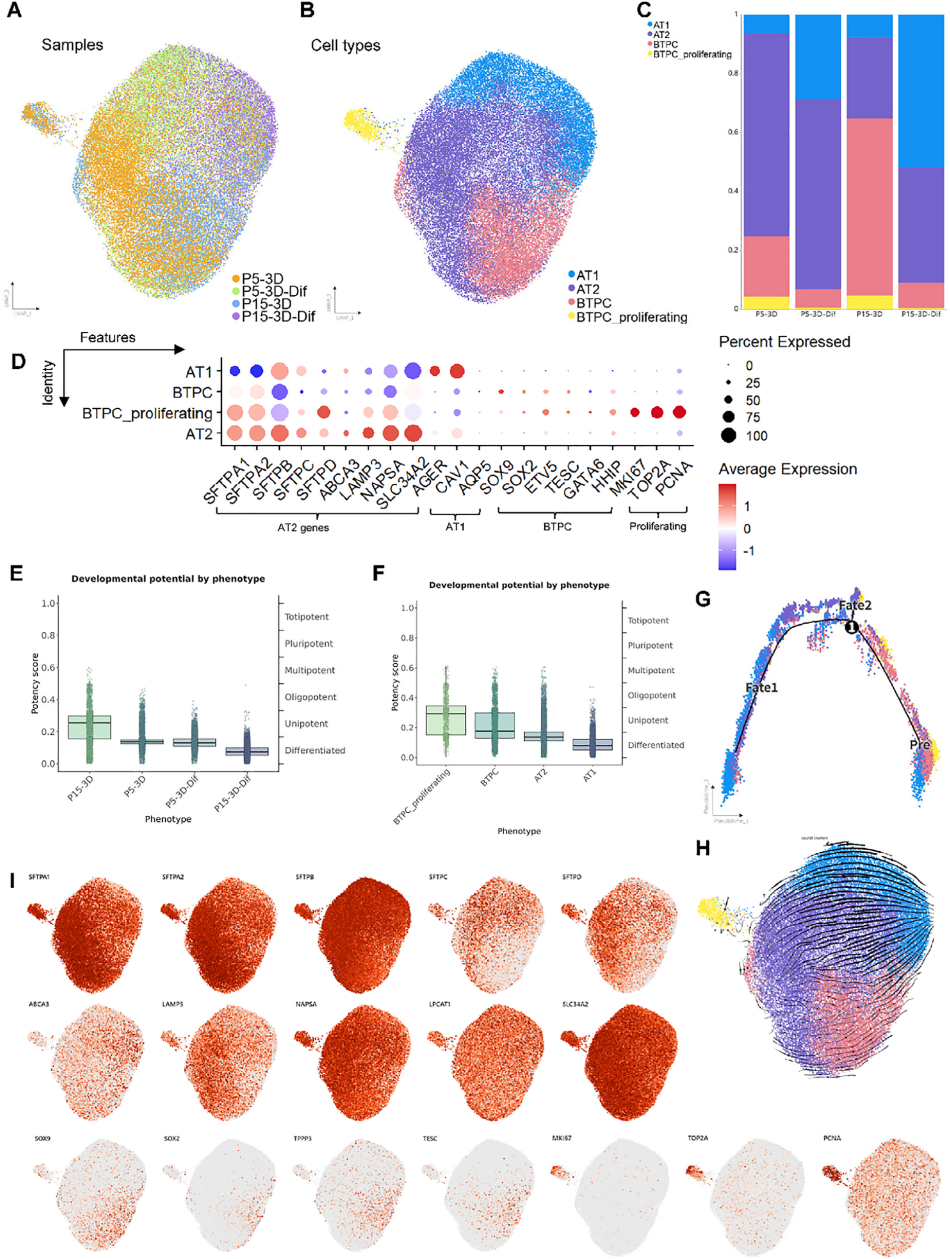
Single‐cell RNA sequencing characterization of alveolar progenitor‐like organoids (APLOs), bud tip‐like organoids (BTLOs), and alveolar‐like organoids (ALOs). (A) Uniform Manifold Approximation and Projection (UMAP) visualization of 43341 single‐cell transcriptomes from APLOs (P5‐3D), BTLOs (P15‐3D), and ALOs (P5‐3D‐Dif and P15‐3D‐Dif). (B) UMAP visualization colored by cell types within the APLC organoids. (C) Compositional analysis showing the relative proportions of identified cell clusters in APLOs (P5‐3D), BTLOs (P15‐3D), and ALOs (P5‐3D‐Dif and P15‐3D‐Dif). (D) Dot plot describing the expression levels of signature genes defining AT2, AT1, lung bud tip progenitor cells, and cellular proliferation across the identified clusters. Dot size represents the percentage of cells expressing the gene within a cluster; color intensity indicates the average expression level. (E) Evaluation of stemness properties across APLOs (P5‐3D), BTLOs (P15‐3D) and ALOs (P5‐3D‐Dif and P15‐3D‐Dif) using a curated stemness gene signature. (F) Comparative stemness assessment of AT2, AT1, BTPC, proliferating BTPC cell clusters. (G) Pseudotime analysis inferring the differentiation trajectory across distinct cell clusters. (H) Analysis of the RNA velocity predicts lineage trajectories in organoid populations. The arrows indicate strong RNA velocities. (I) UMAP plots showing transcript expression of AT2 lineage markers (SFTPA, SFTPB, SFTPC, SFTPD, ABCA3, SLC34A2, LPCAT1, NAPSA, LAMP3), lung bud tip progenitor markers (SOX9, SOX2, TPPP3, TESC) and proliferative markers (MKI67, TOP2A, PCNA) in APLOs (P5‐3D), BTLOs (P15‐3D) and ALOs (P5‐3D‐Dif and P15‐3D‐Dif).

CytoTRACE analysis revealed that BTLOs exhibited the highest stemness scores among all organoid populations, which significantly decreased upon differentiation into ALOs (Figure 4E). Cluster‐level CytoTRACE analysis further demonstrated that proliferating BTPCs and BTPCs possessed the highest stemness potential and were predominantly unipotent, whereas stemness was progressively lost during differentiation toward mature alveolar lineages (Figure 4F). Differential gene expression analysis showed that AT2 cells, compared with BTPCs, displayed significant upregulation of classical AT2‐associated genes (Figure S4A), which were enriched for Gene Ontology Biological Process (GO‐BP) terms related to lipid metabolism and surfactant synthesis (Figure S4B). Gene Ontology Cellular Component (GO‐CC) analysis further indicated enrichment in lamellar body‐associated organelles (Figure S4C).

Pseudotime trajectory analysis revealed that cells originated from both BTPC and proliferating BTPC states, and subsequently bifurcated toward AT2 and AT1 lineages (Figure 4G and Figure S4D). Consistently, RNA velocity analysis using scVelo recapitulated this dynamic alveolar lineage progression, supporting a model in which AT2 cells undergo dedifferentiation toward a BTPC state, followed by redifferentiation into mature AT2 and AT1 cell fates (Figure 4H). This trajectory closely mirrors the developmental progression from embryonic lung bud tip progenitors to mature alveolar cells.

Analysis of key transcription factors demonstrated robust expression of the canonical bud tip progenitor markers SOX2 and SOX9 in BTPCs. During differentiation toward AT2 and AT1 lineages, expression of both transcription factors was progressively downregulated and ultimately silenced (Figure S4E), consistent with exit from the progenitor state.

To further benchmark the developmental relevance of our in vitro system, we integrated scRNA‐seq datasets derived from APLCs (P5‐2D), BTLCs (P15‐2D), and their corresponding APLOs (P5‐3D) and BTLOs (P15‐3D) from two independent donors with a published human fetal lung atlas spanning gestational weeks 5–22 [20]. For integration, 3,000 cells were randomly sampled from each of our datasets and aligned with fetal lung samples containing more than 50 distal epithelial cells. UMAP visualization of the integrated dataset was then generated to assess the developmental positioning of our in vitro‐derived epithelial populations (Figure S5A). Monocle3‐based pseudotime analysis was subsequently performed, with root nodes manually assigned to the Early tip and Early stalk populations. Cells were further stratified into early, middle, and late stages based on pseudotime density distribution (Figure S5,B,D). Cell proportion analysis revealed a progressive increase in early tip cells from P5‐2D to P15‐2D cultures, whereas transition to 3D organoid culture resulted in an increased proportion of late tip cells compared with 2D conditions (Figure S5E).

To further examine progenitor‐associated features, we integrated scRNA‐seq data from APLCs (P5‐2D), BTLCs (P15‐2D), and their derived APLOs and BTLOs from two independent donors with a human fetal lung atlas spanning 15–22 post‐conception weeks [20]. The cultured epithelial populations mapped within the fetal lung epithelial transcriptional landscape (Figure S6,A–D). Compared with APLCs and APLOs, BTLCs and BTLOs showed higher expression of progenitor‐associated genes, including SOX9, SOX2, ID2, and ETV5, and greater transcriptional similarity to fetal distal epithelial and bud tip‐like populations (Figure S6,E,F). This pattern was observed across both donors and in both 2D cultures and their derived 3D organoids.

Furthermore, integration of ALOs derived from multiple donors with published adult AT2 scRNA‐seq datasets [26] demonstrated substantial overlap and shared clustering, accompanied by high‐level expression of canonical adult AT2 markers (Figure S7). To further define the position of differentiated ALO cells within the adult human alveolar epithelium, we projected differentiated ALOs generated from P5 and P15 progenitors from two independent donors onto an adult human alveolar epithelial reference [27] comprising AT1 and AT2 cells. Most ALO cells mapped to the AT2 compartment, whereas a smaller fraction mapped to the AT1 compartment. The transferred cell identities were supported by lineage‐marker expression, with AT1‐assigned cells preferentially expressing AGER and CAV1 and AT2‐assigned cells expressing canonical AT2 markers, including SFTPA1/2, SFTPB, SFTPC, SFTPD, NAPSA, and LAMP3 (Figure S8).

Collectively, these results provide strong evidence that serial passaging of APLCs in 2D culture induces a dedifferentiation process toward a bud tip–like progenitor state, whereas subsequent 3D organoid formation and differentiation promote redifferentiation and maturation into functional alveolar epithelial lineages. This in vitro system therefore recapitulates key developmental transitions from lung bud tip progenitors to mature alveolar cells.

### 2.5 Establishment of an Influenza A Virus Infection Model Using Alveolar‐Like Organoids

Following the development of a stable organoid culture method, we aimed to establish an in vitro model of Influenza A virus (IAV) infection using ALOs. Parallel infection experiments were also conducted with APLOs and BTLOs prior to their differentiation (Figure 5A). To this end, we physically and chemically broke BTLOs and ALOs into pieces to enhance the access of IAV to the apical cell surfaces, followed by IAV inoculation at a multiplicity of infection (MOI) of 1.0 and 0.1.

**FIGURE 5:**
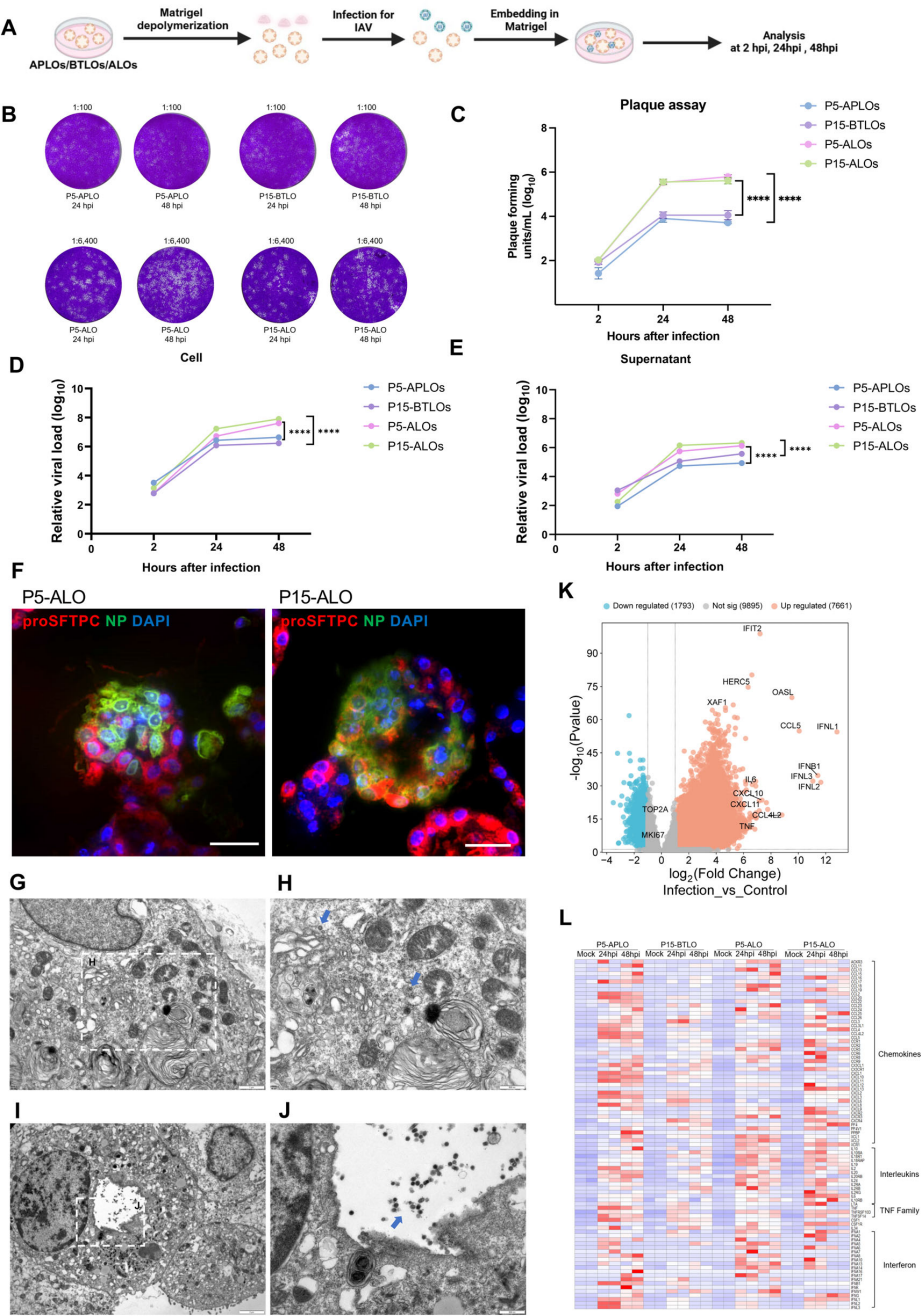
Influenza A virus (IAV) infection of APLOs, BTLOs and ALOs. (A) Schematic diagram outlining the method for IAV infection in APLOs, BTLOs and ALOs. To establish infection models, organoids were collected and broken to expose the luminal space. Then, the pieces of organoids were incubated with IAV at a multiplicity of infection (MOI) of 0.1 or 1.0 for 2 h before resuspended in Matrigel. Cells and supernatants were collected at 2,24,48 h after IAV infection for subsequent experiments. Created in BioRender. Ma, H. (2026) https://BioRender.com/jxmufa7 (B) Representative images for plaque assay using IAV‐infected cells at 24 hpi and 48 hpi. Dilution factors are shown above. (C) Plaque statistics of figure B. Data are presented as mean±SD (n = 3, three plaque assays at each time point).(D) Viral load of IAV in lysed APLOs, BTLOs and ALOs. Data are presented as mean±SD (n = 3, three qPCR assays at each time point). P5‐APLOs, P15‐BTLOs, P5‐ALOs and P15‐ALOs were used in the experiment, ****p < 0.0001. (E) Viral load of IAV in supernatant of APLOs, BTLOs and ALOs. Data are presented as mean±SD (n = 3, three qPCR assays at each time point). P5‐APLOs, P15‐BTLOs, P5‐ALOs and P15‐ALOs were used in the experiment, ****p < 0.0001. (F) Representative images of an infected ALOs at 48hpi. Viral components NP (green) are co‐stained with pro‐SFTPC (red). Scale bars, 25 µm. (G, I) Low‐magnification transmission electron microscopy (TEM) images of ALOs at 48 hpi, showing overall cellular architecture. Scale bars: 1 µm (G), 2 µm (I). **(H, J)** High‐magnification TEM views of infected AT2 cells within ALOs. Blue arrows (H) indicate individual IAV particles dispersed in the cytoplasm. Blue arrows (J) point to viral particles encapsulated within vesicular structures. Scale bars, 500 nm. (K) Volcano plot displaying differentially expressed genes in IAV‐infected ALOs compared to uninfected controls at 48 hpi. (L) Heatmap depicting dynamic transcriptional changes of key immune response genes including chemokines, interleukins, tumor necrosis factor (TNF), and interferon family members in APLOs, BTLOs, and ALOs at 0, 24, and 48 hpi. Expression values are normalized and shown as FPKM.

At different time points after IAV inoculation (MOI of 0.1), virus titer released in the supernatant after organoid infection was detected by plaque forming assay. A significant increase of infectious viral titers in ALOs within 24 h post‐infection (hpi) was observed, indicating the onset of exponential viral replication within this timeframe. Though viral titers were observed to increase along with infection time in APLOs and BTLOs, they were ∼ 100 times lower than that in ALOs (Figure 5,B,C). Consist with the results of plaque forming assay, viral load was significantly higher in both intracellular (cell lysate) and supernatant of ALOs than that of APLOs and BTLOs, suggesting ALOs have higher efficiency of IAV infection and propagation (Figure 5,D,E). In infected ALOs (MOI of 1.0 at 48 hpi), viral particles were clearly observed by IF staining of nucleocapsid protein (NP). These viral components were clearly observed in the cytoplasm of pro‐SFTPC‐expressing cells within ALOs (Figure 5F). To further determine the subcellular consequences of IAV infection, we performed transmission electron microscopy (TEM) analysis. Compared with uninfected ALOs (Figure S9A), the infected ALOs (MOI of 1.0 at 48 hpi) contains viral particles that appeared as electron‐dense objects. The virus particles were characterized by high‐electron‐density ribonucleoproteins surrounded by glycoprotein spikes (Figure 5,G–J).

To investigate transcriptional responses of IAV infection, we performed strand‐specific mRNA sequencing of infected APLOs, BTLOs, and ALOs, respectively. Libraries were prepared from samples harvested at 0, 24, and 48 hpi, with two biological replicates per condition, yielding a total of 24 sequenced libraries. Bulk RNA sequencing analysis of IAV‐infected organoids revealed a pronounced inflammatory response at 24 and 48 h post‐infection compared to uninfected controls. Key inflammatory pathways were significantly upregulated, including genes associated with chemokines, the interleukin family, the TNF superfamily, and interferon signaling. Notably, this transcriptional response was markedly stronger in ALOs than in BTLOs (Figure 5,K,L). Compared to the uninfected control, Gene Ontology (GO) enrichment analysis of upregulated pathways at 24hpi revealed significant activation of immune‐related biological processes, including response to virus, interferon signaling, and chemokine‐mediated inflammatory pathways. By 48 hpi, the dominant enriched processes shifted toward apoptotic signaling and regulation (Figure S9,B,C). Furthermore, weighted gene co‐expression network analysis (WGCNA) demonstrated that, relative to the control group, pathways associated with DNA replication and cell cycle progression were suppressed upon infection, whereas those involved in viral response and immune activation were significantly enhanced (Figure S9,D–F). Of note, the observed innate immune response was entirely cell‐autonomous, as our system is devoid of immune cells, thereby accurately mimicking the initial phase of alveolar infection by IAV. These findings demonstrate that ALOs more effectively recapitulate the pathological changes induced by IAV infection, establishing them as a robust in vitro model for studying IAV pathogenesis.

### 2.6 Engraftment and Differentiation of Alveolar Progenitor‐Like Organoids Following in Vivo Transplantation

To evaluate the in vivo engraftment and differentiation potential of APLOs, we established a bleomycin‐induced lung injury model in immunodeficient mice. Dissociated APLO‐derived cells were delivered intratracheally 3 days after bleomycin administration, and recipient lungs were analyzed at 3, 7, and 21 days post‐transplantation (dpt) (Figure 6A).

**FIGURE 6:**
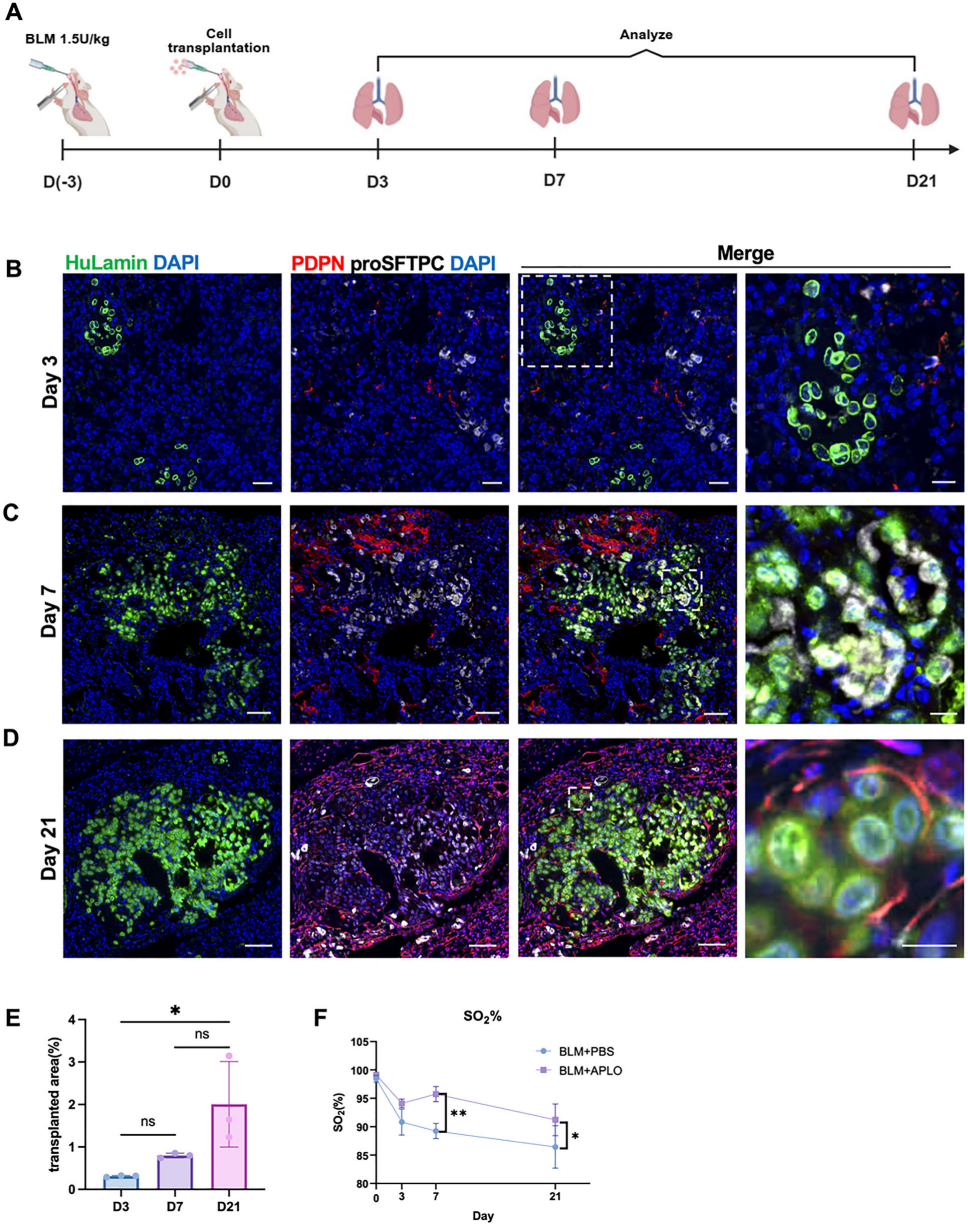
Engraftment and functional assessment of transplanted alveolar progenitor‐like organoids (APLOs) in a murine model of lung injury. (A) Schematic diagram of the experimental timeline for the transplantation and analysis of APLOs in a mouse xenograft model. Created in BioRender. Ma, H. (2026) https://BioRender.com/snq201j (B) Immunofluorescence staining of mouse distal lung sections at 3 days post‐transplantation (3 dpt), showing donor‐derived human cells identified by HuLamin A+C (green) and co‐stained for the pulmonary lineage markers proSFTPC (white) and PDPN (red). Nuclei were counterstained with DAPI. Scale bars, 25 µm (left panels), 12.5 µm (right panel). (C) Immunofluorescence staining of mouse distal lung sections at 7 days post‐transplantation (7 dpt), showing donor‐derived human cells identified by HuLamin A+C (green) and co‐stained for the pulmonary lineage markers proSFTPC (white) and PDPN (red). Nuclei were counterstained with DAPI. Scale bars, 50 µm (left panels), 10 µm (right panel). (D) Immunofluorescence staining of mouse distal lung sections at 21 days post‐transplantation (21 dpt), showing donor‐derived human cells identified by HuLamin A+C (green) and co‐stained for the pulmonary lineage markers proSFTPC (white) and PDPN (red). Nuclei were counterstained with DAPI. Scale bars, 50 µm (left panels), 10 µm (right panel). (E) Quantification of the HuLamin^+^ transplanted area in recipient lungs at 3, 7, and 21 days post‐transplantation. Data are presented as mean ± SD (n = 3). Statistical comparisons were performed among the indicated time points; ns, not significant; *p < 0.05. (F) Arterial blood oxygen saturation (SO_2_%) measured at indicated time points following bleomycin injury in mice treated with APLO‐derived cells or PBS control. Data are presented as mean ± SD (n = 3). *p < 0.05, **p < 0.01.

At 3 dpt, a small number of donor‐derived human cells, identified by HuLamin staining, were detected in distal regions of the injured lung (Figure 6B). Many of the engrafted cells expressed MKI67, and a subset expressed SOX9, indicating that the donor cells retained proliferative and progenitor‐associated characteristics at this early stage (Figure S10A). In contrast, little or no proSFTPC or PDPN expression was detected within the HuLamin‐positive graft regions at this time point (Figure 6B).

By 7 dpt, HuLamin‐positive cells formed larger and more widely distributed clusters within the recipient lungs (Figure 6C and Figure S11). Many donor‐derived cells remained MKI67‐positive and SOX9‐positive (Figures S10B and S11), while proSFTPC expression became detectable in a subset of HuLamin‐positive cells, indicating the emergence of an AT2‐like epithelial phenotype (Figure 6C).

At 21 dpt, the grafts had expanded into larger epithelial clusters composed predominantly of cuboidal HuLamin‐positive cells displaying punctate proSFTPC staining, consistent with an AT2‐like phenotype (Figure 6D). In addition, a small proportion of cells within or along the graft regions exhibited a thinner morphology associated with PDPN expression, suggesting differentiation toward an AT1‐like phenotype. In parallel, MKI67‐positive and SOX9‐positive cells were rarely detected, indicating a transition from the early proliferative progenitor state toward differentiated alveolar epithelial phenotypes (Figure S10C). Quantification of the HuLamin‐positive area showed a progressive increase in transplanted area over time, with a significantly greater graft area at 21 dpt than at 3 dpt (Figure 6E). Thus, APLO‐derived grafts at day 21 consisted predominantly of AT2‐like epithelial clusters together with a smaller population of AT1‐like cells, forming partially integrated reparative alveolar‐like epithelial structures.

To examine the spatial relationship between the engrafted cells and the recipient vasculature, we performed additional CD31 immunostaining. CD31‐positive host endothelial structures were observed in close apposition to, and partially interspersed within, HuLamin‐positive graft regions at both 7 and 21 dpt (Figure S10D), demonstrating a close spatial association between the transplanted epithelial cells and the host pulmonary vasculature.

Peripheral blood oxygen saturation decreased following bleomycin injury in both the PBS‐treated and APLO‐transplanted groups. However, SO_2_ levels were significantly higher in APLO‐transplanted mice than in PBS‐treated controls at 7 and 21 dpt (Figure 6F), indicating that APLO transplantation partially attenuated the decline in blood oxygenation during the observation period.

Collectively, these results indicate that APLO‐derived cells survived, proliferated, and progressively expanded following transplantation into the injured mouse lung. The engrafted cells transitioned from a proliferative progenitor‐associated state toward predominantly AT2‐like and, to a lesser extent, AT1‐like epithelial phenotypes, forming vascular‐associated alveolar‐like epithelial structures accompanied by relative preservation of blood oxygenation.

## 3 Discussion

In this study, we establish a novel and expandable alveolar progenitor‐like cell platform derived from adult AT2 cells, thereby overcoming the long‐standing challenge of maintaining primary AT2 cells during long‐term in vitro culture. By generating bud tip‐like organoids (BTLOs) and directing their differentiation toward alveolar‐like organoids (ALOs), we recapitulate key developmental features of human distal lung epithelium in vitro and produce mature alveolar epithelial cells with functional hallmarks of adult AT2 cells, including lamellar body formation, surfactant production and secretion, uptake of exogenous phospholipids and their trafficking to acidic lamellar body‐like compartments, and the capacity to generate AT1‐like cells. Importantly, ALOs support productive influenza A virus (IAV) infection and mount appropriate early innate immune responses, highlighting their utility as a physiologically relevant platform for investigating viral pathogenesis in the human alveolar compartment. Furthermore, APLO‐derived cells engrafted in injured mouse lungs and gradually acquired predominantly AT2‐like, with limited AT1‐like, epithelial features, providing preliminary evidence of their capacity to contribute to alveolar repair and supporting further evaluation in cell‐based therapeutic studies.

The respiratory system originates from ventral foregut endoderm‐derived buds during early embryogenesis (approximately 4–7 pcw), followed by extensive branching morphogenesis throughout the pseudoglandular stage (5–17 pcw). During the canalicular stage (16–26 pcw), proliferation gradually decreases, and epithelial specialization begins, generating diverse airway lineages including basal, club, goblet, and ciliated cells. Subsequent maturation proceeds through the saccular stage (26–38 pcw) [18, 28, 29], characterized by distal airway dilation and primitive alveolar formation, and culminates in the alveolar stage starting around 38 pcw and extending postnatally into early adulthood, during which secondary septation and alveolar multiplication drive the formation of mature gas‐exchange units [18, 30]. Guided by this developmental blueprint, stepwise differentiation protocols have been established to generate lung epithelial cells from human pluripotent stem cells (hPSCs) by mimicking key developmental signaling pathways [12, 13, 15, 25, 31, 32]. However, PSC‐based approaches often require lengthy, complex differentiation timelines and may be confounded by heterogeneity and safety concerns.

Bud tip progenitor cells represent a central multipotent epithelial population during fetal lung development, capable of giving rise to both proximal airway and distal alveolar lineages. Compared with PSCs, bud tip progenitors offer a more lineage‐restricted intermediate state, enabling a shorter and more deterministic path toward AT2 generation [11] while reducing the risk of off‐target differentiation and malignant transformation after transplantation [14]. Nonetheless, the limited accessibility of fetal tissue and ethical constraints substantially hinder the widespread use of fetal lung‐derived bud tip organoids. Meanwhile, adult AT2‐derived organoids provide valuable in vitro models but typically rely on 3D culture systems, require substantial starting material, and remain difficult to expand at scale, limiting their application in high‐throughput studies and translational development [6, 7].

Building on our previous work showing that chemical reprogramming can induce hepatocyte dedifferentiation into expandable hepatic progenitors [22], we established a culture medium adapted to the properties of adult alveolar epithelial cells. Purified adult AT2 cells could be expanded over multiple passages even when only a small number of starting cells were available. During serial passaging, mature AT2 characteristics gradually decreased, accompanied by the emergence of progenitor‐associated transcriptional features. At late passages, including P15, the expression of markers such as SOX9 became relatively stable, suggesting that adult AT2 cells had transitioned into an expandable progenitor‐like state with features resembling early developmental bud tip‐like cells (BTLCs). This approach allows the long‐term expansion of human alveolar epithelial cells derived from adult tissue while retaining their capacity for subsequent alveolar differentiation. Compared with existing approaches, it may provide several practical advantages, including greater expandability, fewer concerns related to pluripotent stem cell‐derived cells, a relatively direct differentiation process, and the preservation of donor‐specific characteristics.

An important finding of this study is that adult AT2 cells retain considerable developmental plasticity. Previous studies have mainly focused on their ability to differentiate into AT1 cells during alveolar repair. Our results show that, under defined culture conditions, adult AT2 cells can also lose features of mature AT2 cells and acquire a proliferative progenitor‐like state resembling early bud tip cells. These cells can subsequently differentiate toward AT2‐ and AT1‐like epithelial lineages. Thus, the culture system allows the examination of alveolar epithelial transitions in both directions, from mature AT2 cells to a progenitor‐like state and from progenitor‐like cells toward differentiated alveolar epithelium. This may provide a useful model for studying human alveolar development, epithelial changes associated with lung disease, and the cellular processes involved in lung repair.

Upon transfer to 3D culture, expandable APLCs and BTLCs readily formed APLOs and BTLOs, accompanied by further induction of distal epithelial programs. Directed differentiation yielded ALOs with key phenotypic and functional features of mature AT2 cells, including surfactant synthesis and secretion. ALO differentiation was also accompanied by increased expression of AT1‐associated genes, including AGER and CAV1, together with morphological features suggestive of AT1‐like differentiation. However, compared with the AT2 phenotype, the AT1 phenotype was less fully developed, and the expression of some AT1 markers remained distinct from that observed in primary alveolar epithelial cells. These findings suggest that the current differentiation conditions induce AT1‐associated features but may not support complete AT1 maturation. Further studies will therefore be required to determine the extent of AT1 specification and maturation in ALOs. In particular, incorporation of LATS inhibition, as used in a previously reported iAT1 differentiation protocol [33], may help promote AT1 specification and maturation. The BTLO‐to‐ALO transition parallels the in vivo bud tip‐to‐alveolus developmental trajectory, supporting the notion that our culture system reenacts key stages of distal lung epithelial maturation rather than inducing arbitrary lineage conversion.

Beyond its use in studies of alveolar development, our ALO system may also be useful for investigating respiratory viral infections. ALOs supported productive IAV replication and showed early innate immune responses, indicating that they retain relevant features of the human alveolar epithelium during infection. Compared with commonly used transformed cell lines, ALOs maintain polarized epithelial organization, express alveolar markers, and retain the capacity for AT1‐like differentiation. These properties may provide a more tissue‐relevant setting for examining virus–host interactions in the distal lung.

Finally, our in vivo studies showed that APLO‐derived cells could engraft, survive, and expand in the distal regions of bleomycin‐injured lungs. Early proliferative activity was followed by the expression of alveolar epithelial markers, with the grafts consisting predominantly of proSFTPC^+^ AT2‐like epithelial clusters and a smaller proportion of PDPN^+^ AT1‐like cells. However, AT1 maturation and the formation of a thin, well‐integrated alveolar architecture remained limited during the three‐week observation period. Longer‐term studies and further optimization of transplantation conditions will therefore be required to assess graft maturation and structural integration with the host lung.

APLO transplantation was also associated with partial preservation of blood oxygenation. Although SO_2_ declined over time in both groups, consistent with ongoing bleomycin‐induced injury and tissue remodeling, SO_2_ remained higher in the APLO‐transplanted group than in the injury‐only group at later time points. This suggests that APLO transplantation slowed the decline in lung function rather than reversing the underlying disease process. Pulse oximetry reflects the overall physiological state of the lung and does not by itself show that donor‐derived cells formed fully mature gas‐exchanging alveoli. The observed improvement may instead result from several related effects, including the formation of AT2‐like epithelial structures, possible surfactant production, support of damaged alveolar regions, and the close association of grafts with host CD31‐positive vasculature. Intravascular lectin perfusion and direct assessment of alveolar–capillary barrier formation will be important in future studies. Because the engrafted cells occupied only a limited portion of the lung, their local effects were unlikely to fully offset the continuing injury, inflammation, fibrosis, and structural remodeling. Together, these findings suggest that APLO transplantation provides an early and partial functional benefit and support further evaluation of APLO‐derived cells as a potential cell‐based treatment for parenchymal lung diseases such as pulmonary fibrosis.

In conclusion, we show that adult AT2 cells can be expanded in a progenitor‐like state through chemical reprogramming and subsequently differentiated into alveolar epithelial cells with prominent AT2 and additional AT1‐associated features. The resulting ALO system retains key characteristics of human alveolar epithelium and may provide a useful model for studying alveolar epithelial development, respiratory viral infection, and tissue repair. Further studies will be required to assess its relevance to disease modeling and its potential application in translational research.

## 4 Experimental Section

### 4.1 Human Lung Tissue

Human lung tissues were obtained from the Department of Thoracic Surgery at the 905 Hospital of People's Liberation Army Navy. The study was approved by the hospital's Ethics Committee (Approval No. IEC‐AF/29‐4.0) and was conducted in accordance with the Declaration of Helsinki. Written informed consent was obtained from all participants prior to tissue collection.

### 4.2 Human Lung Tissue Dissociation

Pleura was removed, and the remaining human lung tissue washed with PBS containing 1% Antibiotic‐Antimycotic and cut into small pieces. Then samples were digested with 10 mL of enzyme mixture (Collagenase type I: 1.68 mg/ml, Dispase: 5U/ml, DNase: 10U/ml) at 37°C for 1 h with rotation. The cells were filtered through a 70 µm strainer and rinsed with 15 mL DMEM/F12+10% FBS medium through the strainer. The supernatant was removed after centrifugation at 500 g for 10 min, and the cell pellet was resuspended in red blood cell lysis buffer for 10 min, washed with DMEM/F12 containing 10% FBS, and filtered through a 40 µm strainer. Total cells were centrifuged at 500 g for 5 min at 4°C, and the cell pellet was processed for AT2 isolation.

### 4.3 FACS‐Based AT2 Sorting

The single‐cell suspension derived from digested lung tissue was resuspended in FACS buffer (2 mM EDTA and 0.2% fetal calf serum in 1× PBS, pH 7.4) and incubated with HTII‐280 antibody (1:60 dilution) at 4°C for 1 h. The cells were then washed three times with FACS buffer. According to the manufacturer's instructions, the following antibodies were added: PerCP/Cyanine5.5 anti‐human CD31 Antibody, PE anti‐human CD45 Antibody, APC anti‐human CD326 Antibody, and Alexa Fluor 488 anti‐Mouse IgG, IgM (H+L) Secondary Antibody. The cells were incubated at 4°C in the dark for 30 min, then resuspended in FACS buffer, and sorted using a BD Aria III flow cytometer.

### 4.4 AT2 Expansion

The purified AT2 cells were cultured in AEGM (Alveolar Epithelial Growth Medium), which consists of: Advanced DMEM/F12 supplemented with 1× N2 and B27, 1× GlutaMAX, 1×penicillin and streptomycin, 5 µg/mL heparin, and the following growth factors:20 ng/mL EGF,10 ng/mL FGF10, 10 µM Y27632,3 µM CHIR99021,1 µM A8301.The culture medium was refreshed every other day. To assess population growth and doubling time, 10000/cm^2^ cells were seeded on a 10 cm cell culture dish and counted on the designated days, following dissociation with TrypLE Express to give single cells. In each experiment, the cell number was determined based on 3 technical replicates. Population doubling time was calculated using the web tool provided by http://www.doubling‐time.com/compute.php.

### 4.5 Karyotype Analysis

Cultured APLCs at different passages were incubated with 100 ng/mL colchicine at 37°C for 40 min, then washed and dissociated into single cells using trypsin and processed as described above. Karyotyping was performed at the Karyotype analysis department of Hangzhou Kayou Taipu Biotechnology. Chromosomes from at least 40 metaphase‐arrested cells were counted.

### 4.6 Organoids Culture and Differentiation

For APLOs and BTLOs culture, APLCs were resuspended in AEGM and mixed with 5% (v/v) Matrigel, then cultured in suspension in 6‐well plates for 10 days. The medium was changed every other day. For alveolar differentiation, Bud tip‐like organoids were cultured in Alveolar Differentiation Medium (ADM) for 7 days. ADM was based on Advanced DMEM/F12 supplemented with 1× N2 and B27, 1× GlutaMAX, 1× Antibiotic‐Antimycotic, 50 nM Dexamethasone, 0.1 mM cAMP, 0.1 mM IBMX and 10 µM SB431542. The medium was changed every two days.

### 4.7 Live‐Cell Imaging of AT2 Cell Function in Alveolar‐Like Organoids

Differentiated human alveolar‐like organoids were incubated with 1 µM β‐BODIPY FL C12‐HPC (D3792, Invitrogen) in ADM for 24 h at 37°C. Following incubation, the organoids were washed with basal medium and subsequently labeled with 100 nM LysoTracker Red DND‐99 (L7528, Invitrogen) in ADM for 30 min at room temperature. Nuclei were then counterstained with Hoechst 33342 (62249, Thermo Fisher Scientific) for 5 min. After staining, live organoids were imaged using a Leica TCS SP8 STED 3X live‐cell super‐resolution multiphoton laser‐scanning confocal microscope.

### 4.8 Western Blot

Total protein of organoids was extracted using RIPA buffer (P0013B, Beyotime) containing protease inhibitor cocktail (P1010, Beyotime). Samples were sonicated for 30 s on ice, and then centrifuged at 12000xg for 10 min at 4°C. The supernatants were collected and quantified with the BCA Protein Assay Kit (P0012, Beyotime). Quantified protein samples were resolved by 5x SDS‐PAGE (P0015, Beyotime) and transferred to a hydrophobic PVDF transfer membrane (IPVH00010, Merck Millipore). Membranes were blocked in 5% BSA in TBST for 1 h and incubated with primary antibodies overnight at 4°C. Then membranes were washed with TBST three times and incubated for 1 h with secondary antibody at room temperature. The blots were detected using an Enhanced ECL chemiluminescence detection kit (E411‐04, Vazyme) and a digital luminescent image analyzer (BioRad). Primary and secondary antibodies were presented in Supplementary Table S1.

### 4.9 Cells and Viruses

Madin‐Darby canine kidney (MDCK) cells were cultured in DMEM medium supplemented with 10% fetal bovine serum. Influenza H1N1 virus strain A/WSN/1933 (WSN) was propagated in MDCK cells for 3 days at 37°C.

### 4.10 Infection of Organoids with Influenza H1N1 Virus

APLO, BTLOs and ALOs were collected on ice to remove the remaining Matrigel and briefly treated with TrypLE for 5 min to dissociate the organoids before viral inoculation. Cells were then either mock‐infected with infection medium (Advanced DMEM/F12) or challenged with influenza A virus (MOI = 0.1) for 2 h at 37°C. After infection, the virus inoculum was removed, cells were washed with DMEM/F12, resuspended in Matrigel and maintained in the respective organoid medium supplemented with 1 µg/ml TPCK‐treated trypsin for 48 h. The supernatant and cells for plaque assay, bulk RNA‐seq and viral load detection were taken at 2, 24, 48 h post‐IAV infection.

### 4.11 Plaque Formation Assay

MDCK cells in 24‐well plates were washed with PBS twice before inoculated with supernatant collected after organoid infection, and incubated at 37°C for 2 h. After adsorption, remove unabsorbed virus and slowly add the overlay medium (DMEM with 0.75% agarose and 1 µg/mL TPCK‐treated trypsin). After 48 h of incubation, the cells were fixed with 4% paraformaldehyde for 30 min at room temperature and dyed with crystal violet.

### 4.12 Immunofluorescence

Organoids were prepared as described previously. Briefly, organoids were fixed with 4% paraformaldehyde at 4°C for 4 h, and then embedded in paraffin. Sectioned samples (4 µm) were utilized for staining following incubation at 95°C for 15 min for antigen retrieval using 1 mmol Tris‐EDTA. Sections were washed with PBS, incubated with 5% BSA for 20 min at room temperature, followed by primary antibodies at 4°C overnight. Sections were then washed 3 times in PBS, incubated with secondary antibodies for 1 h at room temperature, washed with PBS 3 times, and then added antifade mounting medium with DAPI. Primary antibodies were as follows: Pro‐surfactant protein C (abcam, ab90716, 1:800), E‐cadherin (Proteintech, 20874‐1‐AP, 1:500), SFTPC (ABclonal, A1835, 1:100), HT2‐280 (terracebiotech, TB‐27AHT2‐280, 1:250), NKX2‐1(Beyotime, AG3409, 1:200), Influenza A virus Nucleoprotein (GeneTex, GTX636247, 1:100).

### 4.13 RNA Isolation and Real‐Time qPCR

Total RNA of cells and Organoids were extracted using Eastep Super Total RNA Extraction Kit (Promega) according to the manufacturer's protocols. Real‐time PCR analyses were performed using ABI 7300PLUS and SYBR Green qPCR Mix (Beyotime). Gene transcription was evaluated using the ΔΔCt method normalized to the housekeeping gene GAPDH. Primer sequences are respectively listed in the supplementary information, Table S2.

### 4.14 Bulk RNA Sequencing and Bioinformatic Analysis

Briefly, total RNA was extracted using Trizol Reagent (ThermoFisher, 15596026). The mRNAs were purified using a NEBNext Poly(A) mRNA Magnetic Isolation Module Kit (NEB, E7490) according to the user manual. mRNA libraries were constructed by using the Illumina TrueSeq mRNA sample preparation kit (Cat. No. RS‐122‐2101) according to the manufacturer's instructions. Library sequencing was performed on an Illumina NovaSeq 6000 instrument with 150 bp paired‐end reads.

Adapter sequences and low‐quality reads were trimmed using Fastp (v0.20.0) to obtain high‐quality clean reads. These reads were then aligned to the human reference genome (GRCh38/hg38) with STAR (v2.7.9a). Gene‐level read counts were quantified using featureCounts (v2.0.2) based on the Ensembl human gene annotation (release 104) and used as the mRNA expression profile. Differential expression analysis was performed with DESeq2 (v1.30.1) to calculate fold changes and adjusted p‐values between groups. Functional enrichment analyses, including Gene Ontology and KEGG pathway terms, were conducted using the clusterProfiler R package (v3.18.1) on differentially expressed genes. Alternative splicing events were identified between groups using rMATS (v4.1.1).

### 4.15 Organoid Dissociation and Single‐Cell Suspension Preparation

Cultured organoids were harvested and washed with ice‐cold phosphate‐buffered saline (PBS) to remove residual Matrigel. The organoids were then resuspended in a trypsin‐containing digestion solution and incubated at 37°C for 10 min with continuous agitation.

Digestion was terminated by adding two volumes of DMEM/F12 complete medium. The resulting cell suspension was filtered through a 40 µm cell strainer and centrifuged at 300 × g for 5 min. The supernatant was discarded, and the cell pellet was resuspended in PBS containing 0.5% bovine serum albumin (BSA) for washing. To remove cell aggregates, the suspension was filtered again through a 35 µm cell strainer. Cell viability was assessed using double fluorescence staining with Calcein‐AM (Thermo Fisher Scientific) and Draq7 (BD Biosciences). Validated single‐cell suspensions were subsequently used for single‐cell RNA sequencing library construction.

### 4.16 Single‐Cell RNA Sequencing

The BD Rhapsody system was used to capture the transcriptomic information of the single cells. Single‐cell capture was achieved by random distribution of a single‐cell suspension across >200,000 microwells through a limited dilution approach. Beads with oligonucleotide barcodes were added to saturation so that a bead was paired with a cell in a microwell. The cells were lysed in the microwell to hybridize mRNA molecules to barcoded capture oligos on the beads. Beads were collected into a single tube for reverse transcription and ExoI digestion. Upon cDNA synthesis, each cDNA molecule was tagged on the 5′ end (that is, the 3′ end of an mRNA transcript) with a unique molecular identifier (UMI) and cell barcode indicating its cell of origin. Whole transcriptome libraries were prepared using the BD Rhapsody single‐cell whole‐transcriptome amplification (WTA) workflow, including random priming and extension (RPE), RPE amplification PCR and WTA index PCR. The libraries were quantified using a High Sensitivity DNA chip (Agilent) on a Bioanalyzer 2200 and the Qubit High Sensitivity DNA assay (Thermo Fisher Scientific). All libraries were sequenced by DNBSEQ‐T7 Sequencer (MGI, Shenzhen, China) on a 150 bp paired‐end run.

### 4.17 Single‐Cell RNA Statistical Analysis

scRNA‐seq data analysis was performed by NovelBio Co.,Ltd. with NovelBrain Cloud Analysis Platform. We applied fastp with default parameters, filtering the adaptor sequence, and removed the low‐quality reads to achieve the clean data. To quantify the gene expression of the single‐cell data, we used STARsolo (version 2.7.11b) along with the human genome GRCh38 (ensembl annotation version 104).

### 4.18 Transmission Electron Microscopy

For ultrastructural analysis, APLOs, BTLOs, uninfected and IAV‐infected ALOs (48 hpi) were fixed overnight at 4°C with 2.5% glutaraldehyde in 0.1 M PBS (pH 7.4). Samples were then washed three times in PBS and post‐fixed with 1% osmium tetroxide for 2 h at room temperature. The fixed samples were dehydrated in graded ethanol, transitioned in acetone, and finally embedded. Ultrathin (70 nm) sections were prepared using an ultramicrotome (Leica, EM UC7) and double‐stained with 2% uranyl acetate and lead citrate. Images were captured with a transmission electron microscope (HT7800, Hitachi) at an acceleration voltage of 80 kV.

### 4.19 Animal Experiments

Eight‐week‐old male B‐NDG mice (Biocytogen, Beijing, China) were maintained under specific pathogen‐free conditions at Shanghai Laisenyuan Life Science Co., Ltd. (IACUC: SOP‐MAE‐006‐046), with ambient temperature controlled at 20–26°C, relative humidity at 40–70%, and a 12 h light/dark cycle. All procedures were conducted in accordance with NIH guidelines.

Lung injury was induced via intratracheal instillation of bleomycin (TargetMol, T6116) at a dose of 1.5 U/kg body weight in 30 µL sterile PBS. Three days post‐induction, mice received intratracheal transplantation of 0.5 million APLO‐derived single cells suspended in 40 µL saline. Control animals were administered an equal volume of sterile PBS. At designated time points, mice were euthanized, lung tissues and arterial blood were collected for subsequent analysis.

### 4.20 Statistical Analysis

All data are expressed as mean±SD. Statistical analyses were performed using GraphPad Prism9.0. For comparisons between two groups, a two‐sided Student's t‐test was applied; for comparisons among multiple groups, one‐way ANOVA was used. A p‐value of less than 0.05 was considered statistically significant in all analyses.

## Author Contributions

**Hongqian Ma**: conceptualization, methodology, investigation, formal analysis, project administration, writing – original draft, software, visualization, validation, data curation. **Yuxin Zhang**: methodology, software, data curation, formal analysis, validation, investigation, visualization. **Lujia Sun**: resources, methodology, investigation, writing – original draft, formal analysis, validation, data curation. **Hong Jiang**: resources. **Fan Li**: methodology, investigation. **Wenming Liu**: methodology. **Shenao Zhou**: conceptualization, methodology, writing – review and editing. **Mengting Wang**: methodology. **Weijian Huang**: conceptualization, methodology. **Xu Zhou**: methodology. **Caiyang Chen**: conceptualization. **Qiuqiu Zhang**: methodology. **Xiaoyan Jin**: methodology. **Feihan Yan**: investigation, methodology. **Hongping Wu**: conceptualization. **Yonghong Yin**: resources. **Xinling Wang**: funding acquisition, resources, writing – review and editing. **Hexin Yan**: conceptualization, funding acquisition, writing – review and editing, supervision, project administration, resources. **Weifeng Yu**: funding acquisition, resources, project administration.

## Funding

This work was supported by National Key Research and Development Program of China (2024YFA1107601), the Fundamental Research Funds for the Central Universities (25X010202804), The National Natural Science Foundation of China (82270635 and 82470637), Science and Technology Commission of Shanghai Municipality (23JC1404302 to X.W.), Shanghai Engineering Research Center of Peri‐operative Organ Support and Function Preservation (No. 20DZ2254200) and Shanghai Celliver Biotechnology Co. Ltd.

## Conflicts of Interest

F.L., S.‐A.Z., M. ‐T.W. and Y.‐H.Y. are full‐time employees of Shanghai Celliver Biotechnology Co. Ltd. Shanghai, China. H. ‐X.Y. is co‐founder of Shanghai Celliver Biotechnology Co. Ltd., Shanghai, China and have an equity interest in Celliver Biotechnology Inc. All other authors declare that they have no competing interests.

## Supporting information




**Supporting File**: advs77773‐sup‐0001‐SuppMat.docx.

## Data Availability

All data are available in the main text or the Supporting Information.

## References

[1] A. Egea‐Zorrilla , L. Vera , B. Saez , and A. Pardo‐Saganta , “Promises and Challenges of Cell‐Based Therapies to Promote Lung Regeneration in Idiopathic Pulmonary Fibrosis,” Cells 11, no. 16 (2022): 2595, 10.3390/cells11162595. PMC9406501 36010671

[2] D. van Riel , V. J. Munster , E. de Wit , et al., “Human and Avian Influenza Viruses Target Different Cells in the Lower Respiratory Tract of Humans and Other Mammals,” American Journal of Pathology 171, no. 4 (2007): 1215, 10.2353/ajpath.2007.070248. PMC1988871 17717141

[3] C. Tindle , M. Fuller , A. Fonseca , et al., “Adult Stem Cell‐derived Complete Lung Organoid Models Emulate Lung Disease in COVID‐19,” Elife 10 (2021): 66417, 10.7554/eLife.66417. PMC8463074 34463615

[4] T. J. Desai , D. G. Brownfield , and M. A. Krasnow , “Alveolar Progenitor and Stem Cells in Lung Development, Renewal and Cancer,” Nature 507, no. 7491 (2014): 190–194, 10.1038/nature12930. PMC4013278 24499815

[5] C. E. Barkauskas , M. J. Cronce , C. R. Rackley , et al., “Type 2 Alveolar Cells Are Stem Cells in Adult Lung,” Journal of Clinical Investigation 123, no. 7 (2013): 3025–3036, 10.1172/jci68782. PMC3696553 23921127

[6] J. Youk , T. Kim , K. V. Evans , et al., “Three‐Dimensional Human Alveolar Stem Cell Culture Models Reveal Infection Response to SARS‐CoV‐2,” Cell Stem Cell 27, no. 6 (2020): 905–919.e10, 10.1016/j.stem.2020.10.004. PMC7577700 33142113

[7] H. Katsura , V. Sontake , A. Tata , et al., “Human Lung Stem Cell‐Based Alveolospheres Provide Insights into SARS‐CoV‐2‐Mediated Interferon Responses and Pneumocyte Dysfunction,” Cell Stem Cell 27, no. 6 (2020): 890–904.e8, 10.1016/j.stem.2020.10.005. PMC7577733 33128895

[8] A. A. Salahudeen , S. S. Choi , A. Rustagi , et al., “Progenitor Identification and SARS‐CoV‐2 Infection in human Distal Lung Organoids,” Nature 588, no. 7839 (2020): 670–675, 10.1038/s41586-020-3014-1. PMC8003326 33238290

[9] T. Ebisudani , S. Sugimoto , K. Haga , et al., “Direct Derivation of human Alveolospheres for SARS‐CoV‐2 Infection Modeling and Drug Screening,” Cell Reports 35, no. 10 (2021): 109218, 10.1016/j.celrep.2021.109218. PMC8133488 34038715

[10] M. C. Chiu , C. Li , X. Liu , et al., “A Bipotential Organoid Model of respiratory Epithelium Recapitulates High Infectivity of SARS‐CoV‐2 Omicron Variant,” Cell Discovery 8, no. 1 (2022): 57, 10.1038/s41421-022-00422-1. PMC9203776 35710786

[11] M. M. Lamers , J. van der Vaart , K. Knoops , et al., “An Organoid‐Derived Bronchioalveolar Model for SARS‐CoV‐2 Infection of human Alveolar Type II‐like Cells,” The EMBO Journal 40, no. 5 (2021): 105912, 10.15252/embj.2020105912. PMC7883112 33283287

[12] A. Jacob , M. Morley , F. Hawkins , et al., “Differentiation of Human Pluripotent Stem Cells into Functional Lung Alveolar Epithelial Cells,” Cell Stem Cell 21, no. 4 (2017): 472–488.e10, 10.1016/j.stem.2017.08.014. PMC5755620 28965766

[13] K. M. Abo , J. Sainz de Aja , J. Lindstrom‐Vautrin , et al., “Air‐liquid Interface Culture Promotes Maturation and Allows Environmental Exposure of Pluripotent Stem Cell–derived Alveolar Epithelium,” JCI Insight 7, no. 6 (2022): 155589, 10.1172/jci.insight.155589. PMC8986076 35315362

[14] K. B. McCauley , F. Hawkins , M. Serra , D. C. Thomas , A. Jacob , and D. N. Kotton , “Efficient Derivation of Functional Human Airway Epithelium from Pluripotent Stem Cells via Temporal Regulation of Wnt Signaling,” Cell Stem Cell 20, no. 6 (2017): 844–857.e6, 10.1016/j.stem.2017.03.001. PMC5457392 28366587

[15] A. Jacob , M. Vedaie , D. A. Roberts , et al., “Derivation of Self‐renewing Lung Alveolar Epithelial Type II Cells from human Pluripotent Stem Cells,” Nature Protocols 14, no. 12 (2019): 3303, 10.1038/s41596-019-0220-0. PMC7275645 31732721

[16] M. Z. Nikolić , O. Caritg , Q. Jeng , et al., “Human Embryonic Lung Epithelial Tips Are Multipotent Progenitors That Can be Expanded in vitro as Long‐term Self‐renewing Organoids,” Elife 6 (2017): 26575, 10.7554/eLife.26575. PMC5555721 28665271

[17] A. J. Miller , Q. Yu , M. Czerwinski , et al., “In vitro and In Vivo Development of the Human Airway at Single‐Cell Resolution,” Developmental Cell 53, no. 1 (2020): 117, 10.1016/j.devcel.2020.01.033. PMC7396815 32109386

[18] M. Z. Nikolić , D. Sun , and E. L. Rawlins , “Human Lung Development: Recent Progress and New Challenges,” Development 145, no. 16 (2018): dev163485, 10.1242/dev.163485. PMC6124546 30111617

[19] K. Lim , A. P. A. Donovan , W. Tang , et al., “Organoid Modeling of human Fetal Lung Alveolar Development Reveals Mechanisms of Cell Fate Patterning and Neonatal respiratory Disease,” Cell Stem Cell 30, no. 1 (2023): 20, 10.1016/j.stem.2022.11.013. PMC7618456 36493780

[20] P. He , K. Lim , D. Sun , et al., “A human Fetal Lung Cell Atlas Uncovers Proximal‐distal Gradients of Differentiation and Key Regulators of Epithelial Fates,” Cell 185, no. 25 (2022): 4841, 10.1016/j.cell.2022.11.005. PMC7618435 36493756

[21] K. Lim , E. N. Rutherford , L. Delpiano , et al., “A Novel human Fetal Lung‐derived Alveolar Organoid Model Reveals Mechanisms of Surfactant Protein C Maturation Relevant to Interstitial Lung Disease,” The EMBO Journal 44, no. 3 (2025): 639, 10.1038/s44318-024-00328-6. PMC11790967 39815007

[22] G.‐B. Fu , W.‐J. Huang , M. Zeng , et al., “Expansion and Differentiation of human Hepatocyte‐derived Liver Progenitor‐Like Cells and Their Use for the Study of Hepatotropic Pathogens,” Cell Research 29, no. 1 (2019): 8–22, 10.1038/s41422-018-0103-x. PMC6318298 30361550

[23] R. F. Gonzalez , L. Allen , L. Gonzales , P. L. Ballard , and L. G. Dobbs , “HTII‐280, a Biomarker Specific to the Apical Plasma Membrane of Human Lung Alveolar Type II Cells,” Journal of Histochemistry & Cytochemistry 58, no. 10 (2010): 891–901, 10.1369/jhc.2010.956433. PMC2942742 20566753

[24] M. C. Chiu , C. Li , X. Liu , et al., “A Bipotential Organoid Model of respiratory Epithelium Recapitulates High Infectivity of SARS‐CoV‐2 Omicron Variant,” Cell Discovery 8, no. 1 (2022): 57, 10.1038/s41421-022-00422-1. PMC9203776 35710786

[25] Y. Yamamoto , S. Gotoh , Y. Korogi , et al., “Long‐term Expansion of Alveolar Stem Cells Derived from human iPS Cells in Organoids,” Nature Methods 14, no. 11 (2017): 1097–1106, 10.1038/nmeth.4448. 28967890

[26] K. D. Alysandratos , C. Garcia‐de‐Alba , C. Yao , et al., “Culture Impact on the Transcriptomic Programs of Primary and iPSC‐derived human Alveolar Type 2 Cells,” JCI Insight 8, no. 1 (2023): 158937, 10.1172/jci.insight.158937. PMC9870086 36454643

[27] E. Madissoon , A. J. Oliver , V. Kleshchevnikov , et al., “A Spatially Resolved Atlas of the human Lung Characterizes a Gland‐associated Immune Niche,” Nature Genetics 55, no. 1 (2023): 66–77, 10.1038/s41588-022-01243-4. PMC9839452 36543915

[28] M. Herriges and E. E. Morrisey , “Lung Development: Orchestrating the Generation and Regeneration of a Complex Organ,” Development 141 (2014): 502, 10.1242/dev.098186. PMC3899811 24449833

[29] P. H. Burri , “Fetal and Postnatal Development of the Lung,” Annual Review of Physiology 46, no. 1 (1984): 617–628, 10.1146/annurev.ph.46.030184.003153. 6370120

[30] M. J. Herring , L. F. Putney , G. Wyatt , W. E. Finkbeiner , and D. M. Hyde , “Growth of Alveoli during Postnatal Development in Humans Based on Stereological Estimation,” American Journal of Physiology‐Lung Cellular and Molecular Physiology 307, no. 4 (2014): L338–L344, 10.1152/ajplung.00094.2014. PMC4137164 24907055

[31] S. X. L. Huang , M. D. Green , A. T. de Carvalho , et al., “The in vitro Generation of Lung and Airway Progenitor Cells from human Pluripotent Stem Cells,” Nature Protocols 10, no. 3 (2015): 413–425, 10.1038/nprot.2015.023. PMC4654940 25654758

[32] I. M. Leko , R. T. Schneider , T. A. Thimraj , et al., “A Distal Lung Organoid Model to Study Interstitial Lung Disease, Viral Infection and human Lung Development,” Nature Protocols 18, no. 7 (2023): 2283–2312, 10.1038/s41596-023-00827-6. PMC11486529 37165073

[33] C. L. Burgess , J. Huang , P. S. Bawa , et al., “Generation of human Alveolar Epithelial Type I Cells from Pluripotent Stem Cells,” Cell Stem Cell 31, no. 5 (2024): 657–675.e8, 10.1016/j.stem.2024.03.017. PMC11147407 38642558

