# Supplementary material for: Endowing Flexibility of Alveolar Type II Cells to Recapitulate Development From Lung Bud to Alveoli
Source: Adv Sci (Weinh). 2026 Oct 4:e77773. Online ahead of print. doi: 10.1002/advs.77773 (PMC13634473; doi:10.1002/advs.77773)
Supplement: Supplementary file 1 [file ADVS-9999-e77773-s001.docx]

**List of Supplementary Materials**

Fig. S1. **Generation and characterization of alveolar epithelial progenitor cells (APLCs).**

Fig. S2. **Single-cell RNA sequencing characterization of alveolar epithelial progenitor cells (APLCs).**

Fig. S3. Establishment and characterization of alveolar progenitor-like organoids (APLOs), bud tip-like organoids (BTLOs), and alveolar-like organoids (ALOs).

Fig. S4. **Single-cell RNA sequencing characterization of** alveolar progenitor-like organoids (APLOs), bud tip-like organoids (BTLOs), and alveolar-like organoids (ALOs).

Fig. S5. Determination of developmental stages in APLCs and derived organoids by integrating with the human fetal lung atlas.

Figure S6. Integrated single-cell analysis of APLCs and derived organoids with the human fetal lung atlas to assess developmental positioning and progenitor marker expression.

Fig. S7. Integrated scRNA-seq data of ALOs with Human Adult AEC2.

Figure S8. Integrated single-cell analysis of differentiated ALOs with the adult human alveolar epithelial atlas.

Fig. S9. Influenza A virus (IAV) infection of APLOs, BTLOs and ALOs.

Fig. S10. Engraftment and functional assessment of transplanted alveolar progenitor-like organoids (APLOs) in a murine model of lung injury.

Fig. S11. Engraftment and proliferation of APLO-derived cells in injured mouse lungs.

Table S1. Key resources table.

Table S2. List of primers used for qRT-PCR.

**Fig. S1.** **Generation and characterization of alveolar epithelial progenitor cells (APLCs).** **(A)** Representative flow cytometric gating strategy for the isolation of primary human AT2 cells (hAT2: CD31⁻CD45⁻EpCAM⁺HTII-280⁺) from dissociated human distal lung parenchyma. **(B)** Karyotype analysis confirming a normal diploid chromosome complement in late-passage APLCs (P15) derived from multiple independent donors. **(C)** Immunofluorescence staining of progenitor marker SOX9, and basal cell markers keratin 5 (KRT5) and tumor protein p63 (P63) in APLC-P5 and APLC-P15 samples. Nuclei were counterstained with DAPI. Scale bars, 50 μm. **(D)** Relative mRNA expression levels of proximal airway epithelial markers, including basal cell markers KRT5 and P63, secretory cell marker CC10, and ciliated cell marker FOXJ1, in primary lung tissue and APLCs at different passages, as determined by quantitative real-time PCR. Data are presented as mean ± SD.

**Fig. S2. Single-cell RNA sequencing characterization of alveolar epithelial progenitor cells (APLCs). (A) UMAP plots showing transcriptional expression of AT2 lineage markers SFTPA, SFTPB, ABCA3, SLC34A2, LPCAT1, NAPSA and LAMP3. (B) UMAP plots showing transcriptional dynamics of lung bud progenitor markers SOX9 and SOX2 in** APLC-P5 and APLC-P15. **(C) UMAP plots showing the expression of proximal airway epithelial markers TP63, FOXJ1, and SCGB3A2 in APLCs. Color intensity indicates the relative expression level of each gene.**

**Fig. S3. Establishment and characterization of alveolar progenitor-like organoids (APLOs), bud tip-like organoids (BTLOs), and alveolar-like organoids (ALOs).**

**(A)** Representative bright-field images of APLOs, BTLOs, and ALOs. Scale bars, 200 µm. **(B)** qPCR analysis of AT2 and AT1 marker expression in APLC-P10-2D, P10-APLOs, and P10-ALOs. Data are presented as mean ± SD from three independent experimental replicates using samples derived from a single donor (n = 3). **(C)** qPCR analysis of AT2 and AT1 marker expression in APLC-P15-2D, P15-BTLOs, and P15-ALOs. Data are presented as mean ± SD from three independent experimental replicates using samples derived from a single donor (n = 3). **(D)** Representative immunofluorescence images of P5-ALOs and P15-ALOs stained for AGER (green) and SFTPB (red). Nuclei are counterstained with DAPI (blue). Scale bars, 50 μm. **(E)** Representative fluorescence images showing LysoTracker Red-positive acidic vesicular compartments (red), internalized β-BODIPY FL C12-HPC-labeled phosphatidylcholine (green), and Hoechst-stained nuclei (blue). Scale bars, 5 µm. **(F)** Transmission electron microscopy (TEM) images of P15-BTLOs and P15-ALOs showing lamellar body structures. Scale bars: 2 µm (left), 1 µm (upper right), 500 nm (lower right). **(G)** Immunofluorescence staining of the lung lineage marker NKX2-1 in APLOs, BTLOs, and ALOs. Scale bars, 50 µm.

**Fig. S4. Single-cell RNA sequencing characterization of alveolar progenitor-like organoids (APLOs), bud tip-like organoids (BTLOs), and alveolar-like organoids (ALOs). (A) Volcano plot displaying differentially expressed genes (DEGs) from a direct comparison between the AT2 and BTPC clusters. A total of 443 and 664 genes were significantly enriched in AT2 and BTPCs, respectively (∣log₂FC∣ > 1.19, adjusted P-value < 0.05). (B, C) Gene Ontology (GO) enrichment analysis of biological processes (BP) (B) and cellular components (CC) (C) for genes upregulated in the AT2 cluster compared to BTPCs. (D) Probability density distribution of cells from distinct populations (AT2, AT1, BTPC, and proliferating BTPC) along the inferred pseudotime trajectory. (E) Heatmap depicting the average regulatory activity of key transcription factors across AT2, AT1, BTPC, and proliferating BTPC cell clusters. Each column represents one cell group.**

**Fig. S5. Determination of developmental stages in APLCs and derived organoids by integrating with the human fetal lung atlas. (A)** UMAP visualization of the integrated single-cell RNA sequencing dataset comprising APLCs cultured under 2D conditions (P5-2D and P15-2D), corresponding alveolar progenitor-like organoids (APLOs; P5-3D), bud tip-like organoids (BTLOs; P15-3D) from two independent donors, and a published human fetal lung atlas spanning gestational weeks 5–22. For integration, 3,000 cells were randomly sampled from each *in vitro*-derived dataset and aligned with fetal lung samples containing more than 50 distal epithelial cells. **(B)** Monocle3-based pseudotime trajectory inferred on the integrated UMAP embedding, with root nodes manually assigned to the Early tip and Early stalk populations. Cells are colored according to pseudotime progression. **(C)** UMAP representation of the same dataset with cells categorized into early, middle, and late stages based on pseudotime. **(D)** Density distribution of pseudotime values, showing the stratification of cells into early, middle, and late stages according to pseudotime peaks and boundaries. **(E)** Proportional composition of early, middle, and late tip-stage cells across APLCs, APLOs, BTLOs, and fetal lung epithelial samples from the atlas (gestational weeks 5–22).

**Figure S6. Integrated single-cell analysis of APLCs and derived organoids with the human fetal lung atlas to assess developmental positioning and progenitor marker expression.**

(A) UMAP visualization of the integrated dataset containing human fetal lung reference cells (15, 18, 20, and 22 pcw) , APLCs (P5-2D), BTLCs (P15-2D) and their derived organoids (APLO, BTLO) from two donors. (B) UMAP showing fetal lung reference cells only. (C, D) UMAP showing APLCs (P5-2D), BTLCs (P15-2D) and their derived organoids (APLO, BTLO) from donor 1 and donor 2, respectively. (E) Feature plots showing the expression of representative progenitor markers (SOX9, SOX2, TESC, and TPPP3) in the integrated dataset. (F) Violin plots comparing the expression of progenitor-associated genes (SOX9, SOX2, ID2, ETV5, TESC, and TPPP3) across fetal lung reference samples, APLCs (P5-2D), BTLCs (P15-2D), APLOs and BTLOs.

**Fig. S7. Integrated scRNA-seq data of ALOs with Human Adult Lung Atlas**

**(A-D) UMAP projection of an integrated single-cell RNA sequencing atlas combining *in vitro*-generated ALOs derived from two independent donors and primary adult human alveolar type 2 (AT2) cells from a public reference dataset. Demonstrating close alignment between ALOs and primary AT2 cells. (E) Visualization of canonical AT2 marker genes (e.g., SFTPC, SFTPA1, SFTPA2, LAMP3, ABCA3) on the integrated UMAP coordinates, confirming the acquisition and maintenance of AT2 identity in ALO cultures.**

**Figure S8. Integrated single-cell analysis of differentiated ALOs with the adult human alveolar epithelial atlas.**

**(A)** UMAP visualization of the integrated atlas and differentiated ALO cells, colored by AT1 and AT2 identities. **(B)** UMAP showing the adult atlas cells and ALO cells. **(C)** Projection of ALO cells derived from P5- and P15-derived progenitors from two independent donors onto the atlas, colored by sample identity. **(D)** Proportions of predicted AT1 and AT2 cells in each differentiated ALO sample. **(E)** Dot plot showing the expression of representative AT1 and AT2 marker genes. Dot size indicates the fraction of cells expressing each gene, and color intensity indicates the mean expression level.

**Fig. S9. Influenza A virus (IAV) infection of APLOs, BTLOs and ALOs.**

**(A)** Representative transmission electron microscopy (TEM) images of uninfected ALOs, showing intact ultrastructure. Scale bars: 2 µm (left), 500 nm (right). **(B, C)** Venn diagrams showing numbers of significantly upregulated genes (adjusted p-value < 0.05, log₂FC > 2) 24 hours (B) and 48 hours (C) post-IAV infection compared to uninfected controls, alongside commonly enriched GO biological processes (BP) across APLOs, BTLOs, and ALOs at each time point. **(D)** Weighted gene co-expression network analysis (WGCNA) of all samples pre- and post-infection. Modules significantly correlated with infection are shown; blue and purple heatmaps represent downregulated and upregulated gene modules, respectively. **(E, F)** Functional enrichment analysis of biological processes associated with significantly downregulated (E) and upregulated (F) gene modules post-infection.

**Fig. S10. Engraftment and functional assessment of transplanted alveolar progenitor-like organoids (APLOs) in a murine model of lung injury. (A-C)** Representative immunofluorescence confocal microscopy of lung tissue sections containing donor-derived cells at 3 dpt, 7 dpt and 21 dpt. Sections stained with antibodies detecting HuLamin A+C (green), SOX9 (white), and MKI67 (red). Nuclei stained with DAPI. Scale bars, 50 μm. (D) Representative immunofluorescence images of lung tissue sections at 7 dpt and 21 dpt, stained for HuLamin A+C (green) and CD31 (red). Nuclei stained with DAPI (blue). Right panels represent magnified views of the dashed boxed regions in the left panels. Scale bars, 100 μm (left) and 50 μm (right).

**Fig. S11.** **Engraftment and proliferation of APLO-derived cells in injured mouse lungs.** Representative immunofluorescence images of mouse lung sections analyzed 7 days after intratracheal transplantation of APLOs. The top panel shows a low-magnification overview of multiple engraftment sites within the lung tissue. The white box indicates the region shown in higher magnification in the bottom panels. Sections stained with antibodies detecting HuLamin A+C (green), SOX9 (white), and MKI67 (red). Nuclei stained with DAPI. Scale bars, 200 μm (top) and 50 μm (bottom).

**Table S1. Key resources table.**

| REAGENT or RESOURCE | SOURCE | IDENTIFIER |
| --- | --- | --- |
| Antibodies |  |  |
| Mouse Monoclonal anti-HTII-280 | Terrace Biotech | Cat# TB-27AHT2-280 |
| PE anti-human CD45 Antibody | Biolegend | Cat# 304008, RRID: AB_314396 |
| PerCP/Cyanine5.5 anti-human CD31 Antibody | Biolegend | Cat# 303132, RRID: AB_2566175 |
| APC anti-human CD326 (EpCAM) Antibody | Biolegend | Cat# 324208, RRID: AB_756082 |
| Anti-Prosurfactant Protein C antibody | Abcam | Cat# ab90716, RRID: AB_10674024 |
| E-cadherin Polyclonal antibody | Proteintech | Cat# 20874-1-AP, RRID: AB_10697811 |
| SFTPC Rabbit pAb | ABclonal | Cat# A1835, RRID: AB_2763872 |
| Anti-SOX9 antibody | Abcam | Cat# ab185966, RRID: AB_2728660 |
| Human/Mouse/Rat SOX2 Antibody | R&D systems | Cat# AF2018, RRID: AB_355110 |
| Anti-Ki67 antibody | Abcam | Cat# ab15580, RRID: AB_443209 |
| Thyroid Transcription Factor-1 Rabbit Monoclonal Antibody | Beyotime | Cat# AG3409 |
| Podoplanin Rabbit Monoclonal Antibody | Beyotime | Cat# AG8642 |
| SFTPB Monoclonal Antibody (SPB02 (1B9)) | Thermo Fisher Scientific | Cat# MA1-204, RRID: AB_2633311 |
| Anti-Cytokeratin 5 antibody | Abcam | Cat# ab52635, RRID: AB_869890 |
| p63 antibody | Abcam | Cat# ab124762, RRID: AB_10971840 |
| Influenza A virus Nucleoprotein Antibody | GeneTex | Cat# GTX636247, RRID: AB_2909956 |
| Lamin A antibody [EPR4100] | Abcam | Cat# ab108595, RRID: AB_10866185 |
| Podoplanin Monoclonal Antibody | Thermo Fisher Scientific | Cat# 14-5381-82, RRID: AB_1210505 |
| Anti-CD31 antibody | Abcam | Cat# ab182981, RRID: AB_2920881 |
| Goat Anti-Rabbit IgG H&L (Alexa Fluor® 488) | Abcam | Cat# ab150077, RRID: AB_2630356 |
| Goat anti-Mouse IgG, IgM (H+L) Secondary Antibody, Alexa Fluor™ 488 | Thermo Fisher Scientific | Cat# A-10680, RRID: AB_2534062 |
| Chemicals, Peptides, and Recombinant Proteins |  |  |
| Advanced DMEM/F12 | GIBCO | Cat# 12634028 |
| N2 Supplement (100X) | Basal Media | Cat# S430J4 |
| B27 Supplement (50X) | Basal Media | Cat# S440J7 |
| GlutaMAX | GIBCO | Cat# 35050061 |
| Penicillin and streptomycin (100X) | Basal Media | Cat# S110JV |
| Heparin | TargetMol | Cat# T6531 |
| Fetal Bovine Serum | Corning | Cat# 35-081-CV |
| Human EGF | Novoprotein | Cat# C029 |
| Y27632 | TargetMol | Cat# T1725 |
| CHIR99021 | TargetMol | Cat# T2310 |
| A8301 | TargetMol | Cat# T3031 |
| Dexamethasone | TargetMol | Cat# T1076 |
| 8-Bromo-cAMP sodium salt | TargetMol | Cat# T6747 |
| IBMX | TargetMol | Cat# T1713 |
| SB431542 | TargetMol | Cat# T1726 |
| Matrigel | Corning | Cat# 354230 |
| DNaseⅠ | Thermo Fisher Scientific | Cat# 10104159001 |
| Collagenase type I | GIBCO | Cat# 17100-017 |
| Dispase | Corning | Cat# 354235 |
| LysoTracker™ Red DND-99 | Thermo Fisher Scientific | Cat# L7528 |
| DAPI | Beyotime | Cat# C1005 |
| PBS | Basal Media | Cat# B310KJ |
| Trypsin-EDTA (0.25%) | Basal Media | Cat# S310KJ |
| Trpzyme | Basal Media | Cat# S342KJ |
| HEPES (1M) | Basal Media | Cat# B110JV |
| Trizol | Beyotime | Cat# R0016 |
| 4%PFA | Beyotime | Cat# P0099 |
| BeyoFast™ SYBR Green qPCR Mix (2X) | Beyotime | Cat# D7260 |
| Eastep® Super Total RNA Extraction Kit | Promega | Cat# LS1040 |
| Eastep® RT Master Mix | Promega | Cat# LS2050 |
| β-BODIPY™ FL C12-HPC | Thermo Fisher Scientific | Cat# D3792 |
| Hoechst 33342 | Thermo Fisher Scientific | Cat# 62249 |

**Table S2. List of primers used for qRT-PCR.**

| Species | Gene | Sequence |
| --- | --- | --- |
| Human | SFTPA1_Forward | TGTGTGCGAAGTGAAGGACG |
| Human | SFTPA1_Reverse | CTTTGAGACCATCTCTCCCGT |
| Human | SFTPB_Forward | TGGAGCAAGCATTGCAGTG |
| Human | SFTPB_Reverse | ACTCTTGGCATAGGTCATCGG |
| Human | SFTPC_Forward | CACCTGAAACGCCTTCTTATCG |
| Human | SFTPC_Reverse | TTTCTGGCTCATGTGGAGACC |
| Human | SFTPD_Forward | AAGCAGGGGAACATAGGACCT |
| Human | SFTPD_Reverse | ACACCTCGCTCTCCCTTAGG |
| Human | AGER_Forward | GTGTCCTTCCCAACGGCTC |
| Human | AGER_Reverse | ATTGCCTGGCACCGGAAAA |
| Human | AQP5_Forward | CGGGCTTTCTTCTACGTGG |
| Human | AQP5_Reverse | GCTGGAAGGTCAGAATCAGCTC |
| Human | ABCA3_Forward | CTCCTCTGGAAGAACTACACCC |
| Human | ABCA3_Reverse | GGGCACATTTTCCGACTGAATC |
| Human | NKX2-1_Forward | AGCACACGACTCCGTTCTC |
| Human | NKX2-1_Reverse | GCCCACTTTCTTGTAGCTTTCC |
| Human | SOX2_Forward | TGGACAGTTACGCGCACAT |
| Human | SOX2_Reverse | CGAGTAGGACATGCTGTAGGT |
| Human | SOX9_Forward | AGCGAACGCACATCAAGAC |
| Human | SOX9_Reverse | CTGTAGGCGATCTGTTGGGG |
| Human | ETV5_Forward | TCAGCAAGTCCCTTTTATGGTC |
| Human | ETV5_Reverse | GCTCTTCAGAATCGTGAGCCA |
| Human | ID2_Forward | AGTCCCGTGAGGTCCGTTAG |
| Human | ID2_Reverse | AGTCGTTCATGTTGTATAGCAGG |
| Human | NAPSA_Forward | TCTTCGTACCTCTCTCGAACTAC |
| Human | NAPSA_Reverse | GGCAACAGTGAAGTTTTGTGG |
| Human | LAMP3_Forward | GCGTCCCTGGCCGTAATTT |
| Human | LAMP3_Reverse | TGCTTGCTTAGCTGGTTGCT |
| Human | CAV1_Forward | GCGACCCTAAACACCTCAAC |
| Human | CAV1_Reverse | ATGCCGTCAAAACTGTGTGTC |
| Influenza A virus | Nucleoprotein_Forward | ATGGCGACCAAAGGCACC |
| Influenza A virus | Nucleoprotein_Reverse | CCTCCTCTCGTCAAAAGCAGAG |
| Human | GAPDH_Forward | GGAGCGAGATCCCTCCAAAAT |
| Human | GAPDH_Reverse | GGCTGTTGTCATACTTCTCATGG |
